# Tuning the Softness of the Pendant Arms and the Polyazamacrocyclic Backbone to Chelate the ^203^Pb/^212^Pb Theranostic Pair

**DOI:** 10.1021/acs.inorgchem.3c02610

**Published:** 2024-01-17

**Authors:** Marianna Tosato, Parmissa Randhawa, Luca Lazzari, Brooke L. McNeil, Marco Dalla Tiezza, Giordano Zanoni, Fabrizio Mancin, Laura Orian, Caterina F. Ramogida, Valerio Di Marco

**Affiliations:** † Department of Chemical Sciences, University of Padova, 35131 Padova, Italy; ‡ Radiopharmaceutical Chemistry Section, Nuclear Medicine Unit, AUSL-IRCCS Reggio Emilia, 42122 Reggio Emilia, Italy; § Department of Chemistry, Simon Fraser University, Burnaby, British Columbia V5A 1S6, Canada; ∥ Life Sciences Division, TRIUMF, Vancouver, British Columbia V6T 2A3, Canada

## Abstract

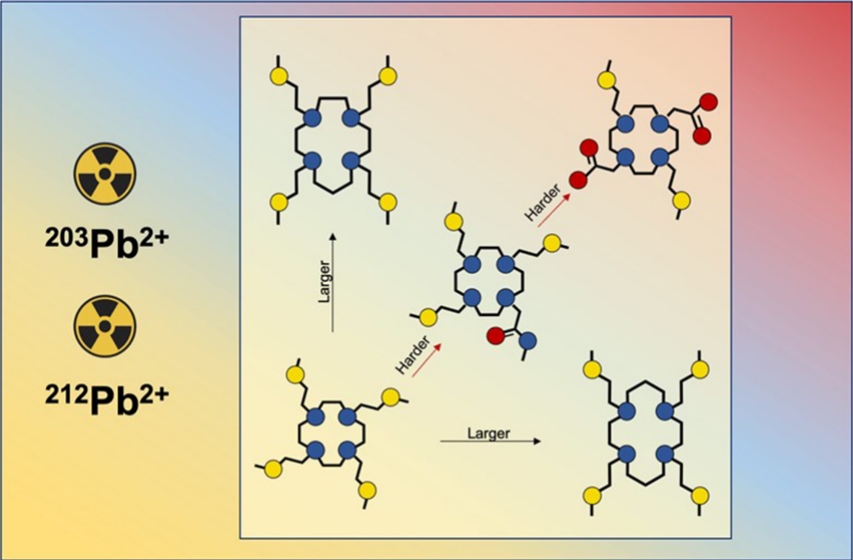

A series of macrocyclic ligands were considered for the chelation of Pb^2+^: 1,4,7,10-tetrakis[2-(methylsulfanyl)ethyl]-1,4,7,10-tetraazacyclododecane (DO4S), 1,4,7-tris[2-(methylsulfanyl)ethyl]-1,4,7,10-tetraazacyclododecane (DO3S), 1,4,7-tris[2-(methylsulfanyl)ethyl]-10-acetamido-1,4,7,10-tetraazacyclododecane (DO3SAm), 1,7-bis[2-(methylsulfanyl)ethyl]-1,4,7,10-tetraazacyclododecane-4,10-diacetic acid (DO2A2S), 1,5,9-tris[2-(methylsulfanyl)ethyl]-1,5,9-triazacyclododecane (TACD3S), 1,4,7,10-tetrakis[2-(methylsulfanyl)ethyl]-1,4,7,10-tetrazacyclotridecane (TRI4S), and 1,4,8,11-tetrakis[2-(methylsulfanyl)ethyl]-1,4,8,11-tetrazacyclotetradecane (TE4S). The equilibrium, the acid-mediated dissociation kinetics, and the structural properties of the Pb^2+^ complexes formed by these chelators were examined by UV–Visible and nuclear magnetic resonance (NMR) spectroscopies, combined with potentiometry and density functional theory (DFT) calculations. The obtained results indicated that DO4S, DO3S, DO3SAm, and DO2A2S were able to efficiently chelate Pb^2+^ and that the most suitable macrocyclic scaffold for Pb^2+^ is 1,4,7,10-tetrazacyclododecane. NMR spectroscopy gave insights into the solution structures of the Pb^2+^ complexes, and ^1^H–^207^Pb interactions confirmed the involvement of S and/or O donors in the metal coordination sphere. Highly fluxional solution behavior was discovered when Pb^2+^ was coordinated to symmetric ligands (i.e., DO4S and DO2A2S) while the introduction of structural asymmetry in DO3S and DO3SAm slowed down the intramolecular dynamics. The ligand ability to chelate [^203^Pb]Pb^2+^ under highly dilute reaction conditions was explored through radiolabeling experiments. While DO4S and DO3S possessed modest performance, DO3SAm and DO2A2S demonstrated high complexation efficiency under mild reaction conditions (pH = 7, 5 min reaction time). The [^203^Pb]Pb^2+^ complexes’ integrity in human serum over 24 h was appreciably good for [^203^Pb][Pb(DO4S)]^2+^ (80 ± 5%) and excellent for [^203^Pb][Pb(DO3SAm)]^2+^ (93 ± 1%) and [^203^Pb][Pb(DO2A2S)] (94 ± 1%). These results reveal the promise of DO2A2S and DO3SAm as chelators in cutting-edge theranostic [^203/212^Pb]Pb^2+^ radiopharmaceuticals.

## Abstract 2

The coordination chemistry of a series of Pb^2+^ complexes formed by several sulfur-containing polyazamacrocycles was investigated by pH-potentiometry, UV−vis, NMR, and DFT calculations. The cyclen-based derivatives, especially when cosubstituted with carboxylates or amides, form the most stable Pb^2+^ complexes. Radiolabeling experiments with [^203^Pb]Pb^2+^ evidenced that some of the studied ligands can incorporate the radioactive Pb^2+^ in high yields and that the corresponding complexes survive in human serum over 24 h.

## 1 Introduction

Targeted radionuclide therapy (TRT) is a rapidly growing strategy for cancer treatment due to its specificity and minimal invasiveness, significantly increasing the quality of life of patients during and after the treatment.^1^ TRT exploits cytotoxic radiation (i.e., α and β^–^ particles or Auger electrons) emitted by radionuclides trapped in a biologically active molecule able to selectively accumulate in cancer cells.^2,3^ An additional benefit of these radiolabeled drugs (i.e., radiopharmaceuticals) is the possibility to recognize the sites of disease, evaluate the therapeutic efficacy, and monitor cancer progression using single photon emission computed tomography (SPECT) or positron emission tomography (PET) as noninvasive imaging techniques, exploiting either γ-rays or annihilation photons generated by the decay of positron (β^*+*^) emitters, respectively.^2,4,5^ The prospect to diagnose and treat cancer using different isotopes of the same element has created the theranostic concept: an emerging clinical management paradigm where the treatment is executed according to an individually tailored therapeutic regime.^2,4,5^

α-Radiation is characterized by a high linear energy transfer (50–230 keV/μm) and a short-range emission (<10 cellular diameters).^6,7^ Therefore, if compared to β^–^ particles, α-particles produce more lethal DNA double-strand breaks per radiation track when traversing the cell nucleus, while their short emission range increases the safety profile of the radiopharmaceutical because unwanted irradiation around the target cells is greatly reduced or absent.^7,8^ Additionally, the cytotoxicity of the α-emitters is independent of oxygen concentration, dose rate, and cell cycle position.^8^ Collectively, these features make α-emitters ideal for the treatment of single tumor cells, micrometastases, lymphatic and vascular cancer cells (e.g., lymphoma and leukemia), or in the case of residual disease after surgical debulking, whereas β^–^-emitters are better for the eradication of macroscopic tumors.^7^

However, most of the α-emitters presently under preclinical and/or clinical investigations, such as actinium-225 (*t*_1/2_ = 9.92 days), astatine-211 (*t*_1/2_ = 7.2 h), bismuth-212/213 (*t*_1/2_ = 60.6/45.6 min), and thorium-226/227 (*t*_1/2_ = 30.6 min/18.70 days), have a lack of diagnostic isotopes, thus forcing the use of chemically different nuclides as imaging surrogates.^9,10^ A drawback of this “different-element” theranostic approach is the possible discrepancy in the biodistribution of the corresponding radiolabeled compound as a result of the different element chemistry.

In this context, a rare opportunity of an α-emitter possessing an imaging counterpart is represented by two lead radioisotopes, lead-203 (^203^Pb) and lead-212 (^212^Pb).^11^ The former (*t*_1/2_ = 51.9 h, *E*_γ_ = 279.1 keV, *I*_γ_ = 81%) is suitable for SPECT imaging as it releases γ-photons during its decays via electron capture to the ground state of thallium-203.^5,11^ On the other hand, ^212^Pb purely decays by β^–^-emission (*t*_1/2_ = 10.6 h, *E*_β_^–^ = 100 keV, *I*_β_^–^ = 100%), but it is an *in vivo* α-particle generator through its decay daughter ^212^Bi (*E*_α_ = 6.3 MeV, *I*_α_ = 36%).^5,12^ Remarkably, the use of ^212^Pb instead of ^212^Bi allows the short half-life of the latter to be circumvented, which not only poses a logistical dilemma for radiolabeling and drug administration but also limits the time frame for the circulation and the accumulation of the radiopharmaceutical in the malignant target site.^7^ Moreover, the *in vivo*^212^Pb/^212^Bi generator allows the delivery of up to 10 times more doses per unit of administered activity compared to ^212^Bi alone or the α-emitter ^213^Bi.^9,13^

However, to establish the routine use of ^203/212^Pb in the clinic, different challenges must be addressed.^14−17^ Both radionuclides must be delivered with high specificity and retained within the vicinity of the biological target.^2,4^ This can be accomplished by the formation of a stable radiometal complex with a chelator in turn covalently appended to a tumor-targeting moiety.^2,4,18−20^ An ideal chelating agent must possess high thermodynamic stability and kinetic inertness toward Pb^2+^ to minimize undesired dissociation, transchelation, and transmetalation reactions in biological environments.^21^ Otherwise, any released radiometal would be taken up in nontarget organs, thus resulting in high background activity levels, limiting the target visualization, and posing an undesirable radiation burden on healthy sites.^22,23^ Fast and quantitative complexation, possibly under mild reaction conditions (i.e., room temperature, neutral pH), is also necessary to preserve the activity and allow the use of heat and/or pH-sensitive biovectors.^2−4,22^ Moreover, the chelating ligand can be used to modify the pharmacokinetics of the radiopharmaceutical, in particular when small molecules or peptides are used.^2,4,5^

**Figure 1:**
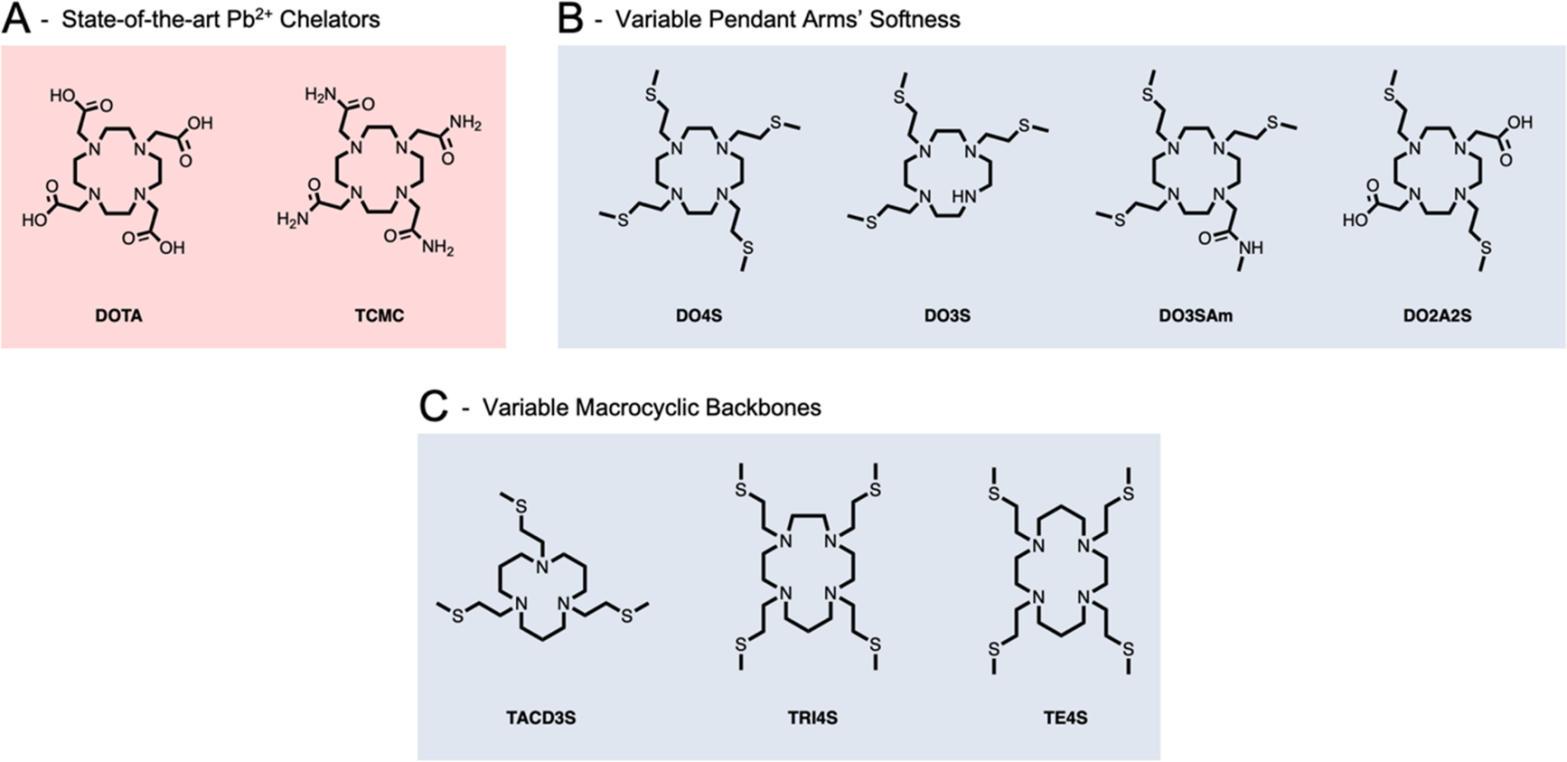
(A) State-of-the-art ligands for [^203/212^Pb]Pb^2+^ chelation (DOTA and TCMC) and ligands investigated in this work having (B) different pendant arms’ softness (DO4S, DO3S, DO3SAm, and DO2A2S) and (C) different macrocyclic backbones (TACD3S, TRI4S, and TE4S).

To date, the chelation of [^203/212^Pb]Pb^2+^ has been predominantly explored with 1,4,7,10-tetraazacyclododecane-1,4,7,10-tetraacetic acid (DOTA) and its tetracetamide derivative 1,4,7,10-tetraaza-1,4,7,10-tetra-(2-carbamoylmethyl)-cyclododecane (TCMC or DOTAM) (Figure 1 - **A**).^5,24^ However, DOTA is not able to retain the daughter isotope ^212^Bi formed during the decay of ^212^Pb, causing off-target toxicity, primarily in the kidneys.^5,24^ Moreover, DOTA is susceptible to acidic conditions which can result in the acid-promoted dissociation of the radiometal cation.^5^ The substitution of the carboxylic donors of DOTA with amide groups, giving TCMC, has improved the kinetic inertness of Pb^2+^ complexes, but the destabilization of the decay daughter from the TCMC complex remains an issue.^5^ Despite the great potential of ^203/212^Pb, a proper ligand that can bind Pb^2+^ strongly and efficiently *in vivo* is still sought, and it is one of the keys to boosting the advancement of [^203/212^Pb]Pb^2+^ toward the clinic.^25^

In this context, inspired by the improvement achieved by swapping the carboxylic groups of DOTA with amide pendants in TCMC, we have hypothesized that the introduction of softer arms could further improve the performance of the generated complexes as they could optimally complement the borderline-soft nature of Pb^2+^.^26^ Hence, we have considered a class of S-bearing chelators previously developed by our group as multipurpose ligands capable of trapping borderline-soft metals such as Ag^+^, Cd^2+^, Cu^2+^, and Cu^+^.^2,4,27−29^ The ligands investigated hereby are 1,4,7,10-tetrakis[2-(methylsulfanyl)ethyl]-1,4,7,10-tetraazacyclododecane (DO4S), 1,4,7-tris[2-(methylsulfanyl)ethyl]-1,4,7,10-tetraazacyclododecane (DO3S), 1,4,7-tris[2-(methylsulfanyl)ethyl]-10-acetamido-1,4,7,10-tetraazacyclododecane (DO3SAm), 1,7-bis[2-(methylsulfanyl)ethyl]-1,4,7,10-tetraazacyclododecane-4,10-diacetic acid (DO2A2S) (Figure 1 - **B**), 1,5,9-tris[2-(methylsulfanyl)ethyl]-1,5,9-triazacyclododecane (TACD3S), 1,4,7,10-tetrakis[2-(methylsulfanyl)ethyl]-1,4,7,10-tetrazacyclotridecane (TRI4S), and 1,4,8,11-tetrakis[2-(methylsulfanyl)ethyl]-1,4,8,11-tetrazacyclotetradecane (TE4S) (Figure 1 - **C**).^27,28^ The chelators resumed in Figure 1 - **B** are based on the same 1,4,7,10-tetrazacyclododecane (cyclen) backbone, varying in the nature of the pendant arms which modulate their chemical softness. Moreover, some of these ligands (i.e., DO3SAm and DO2A2S) mimic the bifunctional version of the pure S-containing derivatives. The ligands shown in Figure 1 - **C** differ by the ring scaffolds which are no longer based on cyclen.^27,28^

In the present work, we detail the evaluation of the macrocycles reported in Figure 1 - **B, C** for the chelation of Pb^2+^ radioisotopes aiming to investigate the impact of the structural changes (i.e., pendant arms and macrocyclic backbones) on the properties of the corresponding complexes. For this purpose, their solution thermodynamics, formation, and acid-mediated dissociation kinetics were assessed with nonradioactive Pb^2+^ through a combination of UV–Visible (UV–Vis) spectrophotometric, pH-potentiometric, and nuclear magnetic resonance (NMR) experiments. Density functional theory (DFT) calculations were conducted as well to further support the experimental data. The solution structures of the Pb^2+^ complexes were explored using variable-pH and variable-temperature monodimensional and bidimensional NMR. Radiolabeling was then performed with [^203^Pb]Pb^2+^ to evaluate the complexation efficiency of the investigated chelators under extremely dilute reaction conditions. Additionally, the human serum integrity of the corresponding [^203^Pb]Pb^2+^ complexes was probed to fully assess the suitability of these chelating agents for [^203/212^Pb]Pb^2+^-based theranostic radiopharmaceuticals. To the best of our knowledge, this work represents the first example of polyazamacrocyclic ligands bearing sulfanyl pendant arms proposed for the complexation of Pb^2+^ radioisotopes.

## 2 Results and Discussion

### 2.1 Probing the Formation of Pb^2+^ Complexes

The formation of the Pb^2+^ complexes was qualitatively assessed at room temperature via UV–Vis and ^1^H NMR spectroscopies. This is an initial necessary step to be conducted as the thermodynamic investigations require a knowledge of the time to reach the equilibrium conditions.

The electronic spectra and the variation of the absorbance over time for the Pb^2+^ complexation reactions at different pH with the cyclen-based ligands (DO4S, DO3S, DO3SAm, and DO2A2S) are illustrated in Figures S1–S3, while the complexation times are summarized in Table S1. No complex formation was detected below pH = 4 with the pure S-containing ligands DO4S and DO3S, as no spectral changes were detected with respect to the free chelators, neither after 2 weeks at room temperature nor after prolonged heating (Figure S1). At pH > 4, complex formation occurred but it was rather slow: for example, at pH = 5 and in the presence of equimolar ligand and metal concentrations (10^–4^ M), equilibrium was reached in 24 h with DO4S and 6 h with DO3S (Figure S2). The presence of O donors in the ligand’s pendant arms sped up the complexation kinetics (Table S1). For example, at pH = 5 the reaction with DO3SAm occurred in 1 h (Figure S2) and that with DO2A2S was practically instantaneous.

These results can be interpreted by considering the different charges of the pendant arms. The favorable Coulombic interactions between Pb^2+^ and the O atoms can increase the local concentration of the metal ion near the donors due to the formation of out-of-cage intermediates which are afterwards transformed into the final in-cage products.^4^ For DO2A2S, this effect is more pronounced than for DO3SAm as in the latter case the O atoms are only partially negatively charged. No out-of-cage intermediates can be formed by the thioether side chains as they are not negatively charged.

For all chelators, the reaction speed increased markedly with the pH (for example, the reaction time of DO4S dropped from 24 h at pH = 5 to 1 h at pH = 7.4, Figure S3). This can be explained by considering the decrease in the proton content on the ligand when the pH is increased, which generates progressively less intense electrostatic repulsions between the cation and donors located in the macrocyclic ring, as also previously evidenced with other 2+ cations such as Cu^2+^.^4,28^

Noncyclen-based ligands (i.e., TACD3S, TRI4S, and TE4S) were demonstrated to not be able to complex Pb^2+^ at pH < 7, as the ^1^H NMR spectra of the metal–ligand mixtures at various pH were identical to the spectra of the free ligands (Figure S4). The stability drop with respect to the cyclen-based ligands can be attributed to the increased size of the macrocyclic scaffolds of TRI4S and TE4S and to the increased spacer between the N donors in TACD3S. These structural changes may cause worse matching between the size of Pb^2+^ and the ring cavities. Such data agrees with the binding affinity trend observed with the corresponding nonfunctionalized macrocycles (log*K*_Pb^2+^__-cyclen_ = 15.9, log*K*_Pb^2+^__-cyclam_ = 13.48, log*K*_Pb^2+^__-13aneN4_ = 10.83 at *T* = 25 °C).^30^ As high thermodynamic stability is a paramount requirement for a ligand in metal-based radiopharmaceuticals, the subsequent equilibrium and kinetic investigations were focused on the cyclen-based chelators reported in Figure 1 - **B**.

### 2.2 Solution Thermodynamics of Pb^2+^ Complexes

The rather slow kinetics of the complexation reactions with DO4S, DO3S, and DO3SAm prevented the use of conventional in-cell methods to determine the speciation and the stability constants (log β) of the corresponding Pb^2+^ complexes. Out-of-cell UV–Vis spectrophotometric titrations were therefore employed. With DO2A2S, the equilibrium was reached quickly enough so that additional direct in-cell potentiometric measurements were conducted.

The electronic spectra of the investigated Pb^2+^-ligand pairs at equilibrium at different pH are reported in Figure S5, while the spectroscopic data are summarized in Table S2. The variation of the absorbance at the characteristic wavelength as a function of pH is shown in Figure S6.

Significant spectroscopic changes can be observed when Pb^2+^ is added to the ligand solutions (Figure S5), as an intense band in the UV-B/near-UV spectral regions appeared (the spectra of the free ligands are reported in our previous works^27,28^): these signals are therefore diagnostic of the complexation event. DO4S, DO3S, and DO3SAm coordinate Pb^2+^, forming a mononuclear complex with the ligand in its totally deprotonated form (as shown in Figure 1 - **B**), namely, [PbL]^2+^. On the other hand, for DO2A2S, the monoprotonated species [PbLH]^+^ was also found. The speciation model of Pb^2+^-DO2A2S was further confirmed by pH-potentiometric titrations.

**Figure 2:**
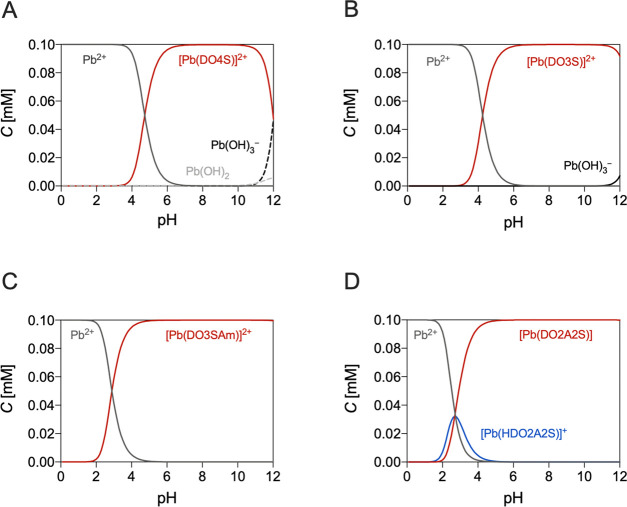
Distribution diagrams of (A) Pb^2+^-DO4S, (B) Pb^2+^-DO3S, (C) Pb^2+^-DO3SAm, and (D) Pb^2+^-DO2A2S (*C*_Pb_^2+^ = *C*_ligand_ = 1.0 × 10^–4^ M).

The obtained formation constants (log β) are presented in Table 1, and the corresponding speciation diagrams are shown in Figure 2.

**Table 1:** Overall Stability Constants (log β) and pPb Values for the Pb^2+^ Complexes with the Investigated Cyclen-Based S-Containing Ligands at *I* = 0.15 M NaNO_3_ and *T* = 25 °C^a^

**Ligand**	**Equilibriumreaction**^b^	**log** **β**	**pPb**^d^	pPb*^e^
DO4S	Pb^2+^ + L ⇋ [PbL]^2+^	12.3 ± 0.1	10.2	10.0
DO3S	Pb^2+^ + L ⇋ [PbL]^2+^	14.2 ± 0.1	11.3	11.0
DO3SAm	Pb^2+^ + L ⇋ [PbL]^2+^	16.8 ± 0.1	14.2	13.9
DO2A2S	Pb^2+^ + H^+^ + L^2–^ ⇋ [PbHL]^+^	20.89 ± 0.07^c^	15.7	15.4
	Pb^2+^ + L^2–^ ⇋ [PbL]	18.2 ± 0.1^c^/18.3 ± 0.1		

a Unless otherwise stated, the log β values were obtained by UV–Vis spectrophotometric titrations.

b L denotes the ligand in its totally deprotonated form as shown in Figure 1 - **B**.

c Obtained by pH-potentiometry.

d pPb = −log[Pb^2+^]_free_ calculated at *C*_Pb_^2+^ = 10^–6^ M, *C*_ligand_ = 10^–5^ M, and pH = 7.4.

e pPb* = −log([Pb^2+^] + [PbOH^+^] + [Pb(OH)_2_] + [Pb(OH)_3_^–^] + [Pb_2_(OH)^3+^] + [Pb_3_(OH)_4_^2+^] + [Pb_4_(OH)_4_^4+^] + [Pb_6_(OH)_8_^4+^]) calculated at *C*_Pb_^2+^ = 10^–6^ M, *C*_ligand_ = 10^–5^ M, and pH = 7.4.

To compare the chelating ability among the investigated ligands and with the state-of-the-art DOTA and TCMC, it is necessary to consider their different acidity constants because protonation reactions and metal complex formation are competitive processes. To do so, the pPb values were computed (defined either as pPb = −log[Pb^2+^]_free_ or as pPb* = −log([Pb^2+^] + [PbOH^+^] + [Pb(OH)_2_] + [Pb(OH)_3_^–^] + [Pb_2_(OH)^3+^] + [Pb_3_(OH)_4_^2+^] + [Pb_4_(OH)_4_^4+^] + [Pb_6_(OH)_8_^4+^])): the higher the pPb, the greater the stability of the considered complex.^31^ The obtained values are detailed in Table 1.

The pPb values demonstrate that the investigated chelators form weaker complexes if compared with DOTA and TCMC, as pPb values computed for the two latter compounds (pPb_DOTA_ = 20 and pPb_TCMC_ > 18) are higher than those reported in Table 1.^32,33^ On the other hand, the Pb^2+^ complexes formed with the two O-containing ligands (i.e., DO3SAm and DO2A2S) possess higher pPb values than those formed by DO4S and DO3S (pPb_DO2A2S_ = 15.7 and pPb_DO3SAm_ = 14.2 vs pPb_DO4S_ = 10.3 and pPb_DO3S_ = 11.3).

This pPb trend indicates that the presence of S-containing arms reduces the stability of the complex and also that the O donors form more stable complexes than the S ones. These results are surprising for Pb^2+^, which is regarded to be a borderline-soft cation rather than a hard one.^26^ They also are opposite to those previously detected for the same chelators with other borderline and soft cations such as Ag^+^, Cd^2+^, and Cu^+^, for which the number of S-containing arms positively correlated with the stability of the resulting complexes.^2,4,27^

**Figure 3:**
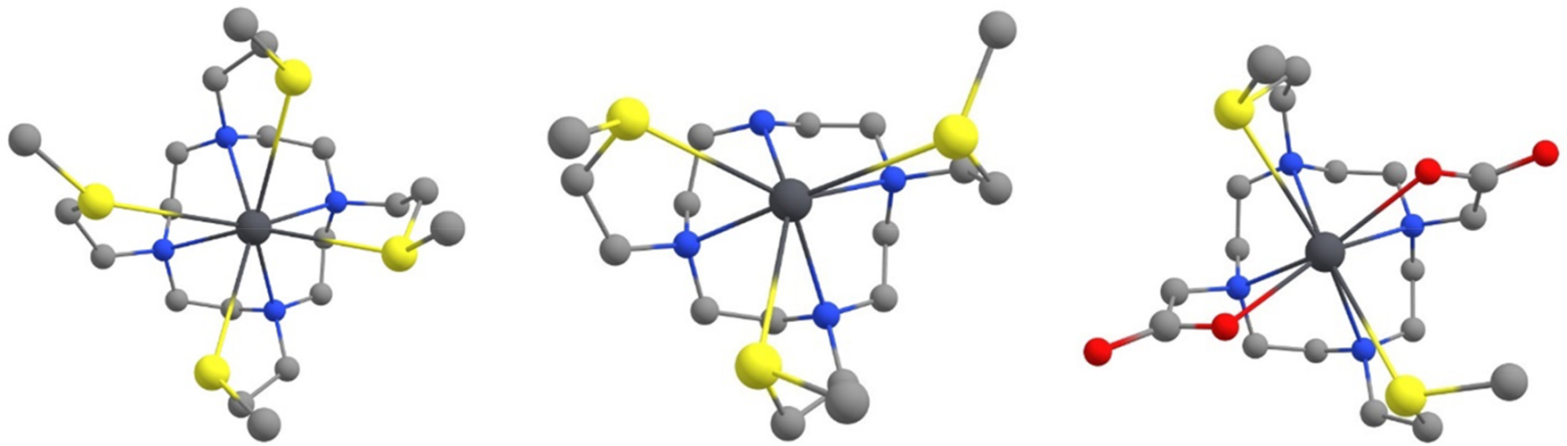
DFT-optimized structures of the complexes formed between Pb^2+^ and DO4S, DO3S, and DO2A2S. Level of theory: (COSMO-)ZORA-OPBE/TZ2P//ZORA-OPBE/TZP.

A DFT analysis was carried out to further corroborate that DO2A2S forms Pb^2+^ complexes with higher stability than DO4S; DO3S was also added to the calculation to check if our computational protocol can reproduce the experimentally found thermodynamic stability order. The electronic (Δ*E*) and Gibbs free energies (Δ*G*) in the gas phase and water have been calculated. As reported in Table 2, the greater the number of S donors coordinated to the metal center, the lower the stability of the resulting complexes, as experimentally observed. For example, Pb^2+^-DO2A2S showed a marked stabilization of 36.6 kcal/mol in water when compared to the complexes formed with the pure S-containing analogue DO4S. The DFT analysis also allowed the determination of the structures of these Pb^2+^ complexes, which are shown in Figure 3.

**Table 2:** Electronic and Gibbs Free Energies (in Gas Phase and in Water) for the Pb^2+^ Complexes Formed with the Investigated S-Rich Cyclen-Based Ligands^a^

		**Gas phase**		**Water**	
**Ligand**	**Coordination mode**	**Δ*****E*** [kcal/mol]	**Δ*****G*** [kcal/mol]	**Δ*****E*** [kcal/mol]	**Δ*****G*** [kcal/mol]
DO4S	N_4_S_4_	–234.2	–218.1	–60.5	–44.4
DO3S	N_4_S_3_	–235.4	–218.6	–69.0	–52.2
DO2A2S	N_4_O_2_S_2_	–509.7	–497.2	–93.5	–81.0

a Level of theory: (COSMO-)ZORA-OPBE/TZ2P//ZORA-OPBE/TZP.

### 2.3 Solution Structures of Pb^2+^ Complexes

**Figure 4:**
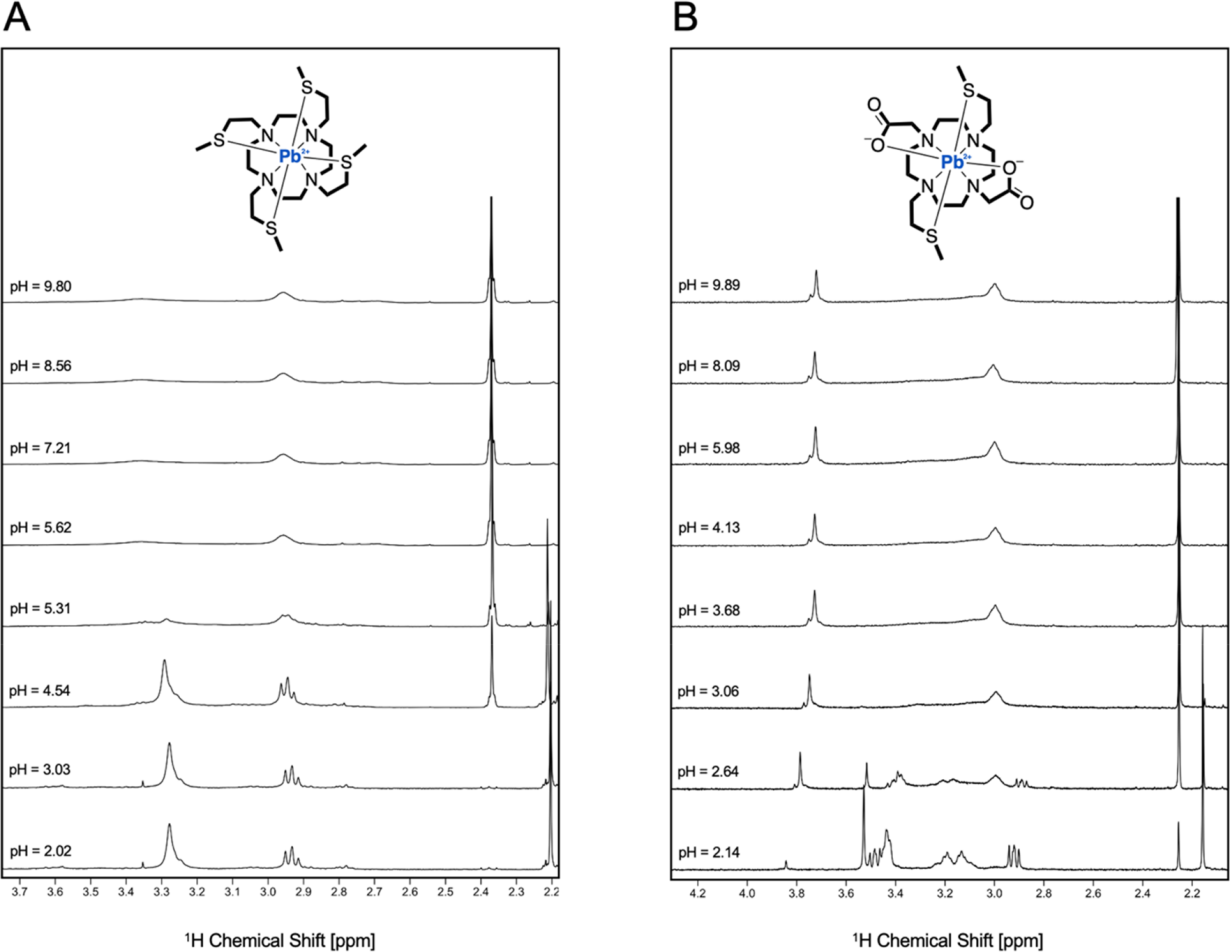
^1^H NMR spectra of (A) Pb^2+^-DO4S and (B) Pb^2+^-DO2A2S at different pH values (400 MHz, *T* = 25 °C, 90% H_2_O + 10% D_2_O, and *C*_Pb_^2+^ = *C*_ligand_ = 1.0 × 10^–3^ M).

Variable-pH ^1^H NMR titrations were conducted to gain experimental insight into the solution structure of the Pb^2+^ complexes formed by the sulfanyl-containing cyclen-based ligands and to support the speciation models obtained by UV–Vis spectroscopy and potentiometry. The ^1^H NMR spectra at different pH are collected in Figure 4 and Figure S7. The spectral assignations are summarized in Table S3, based on bidimensional spectra reported in Figures S8–S10.

The comparison between the spectra of the free chelators (reported in our previous work^27^) and the Pb^2+^-ligand mixtures undoubtedly demonstrated the complexation event: significant modifications in both the chemical shifts and the coupling patterns were detected, as exemplified in Figures S11 and S12. All of the signals experienced a downfield shift upon complexation as a consequence of the ligand-to-metal electron density donation. This, combined with the Pb^2+^ coordination number preferences, suggests that both the cyclen core and the pendant arms are interacting on average with the metal ion. In the pH range where the nonquantitative formation of the Pb^2+^ complexes was predictable from the speciation diagrams shown in Figure 2, the ^1^H NMR spectra always appeared as the convolution of those of the free ligands and the complexes (Figure 4 and Figure S7). This spectral characteristic indicates the absence of ligand-to-complex dynamic exchange processes occurring on the NMR time scale.

With DO4S (Figure 4 - **A**) and DO3S and DO3SAm (Figure S7), no proton content dependency of the ^1^H resonances was observed, indicating that a single species exists at equilibrium, in agreement with the potentiometric and spectrophotometric data (i.e., [PbL]^2+^, *vide supra*). On the other hand, for Pb^2+^-DO2A2S, the resonance of the methylene groups belonging to the acetate pendants experienced a shift toward lower ppm with the pH increases, as reported in Figure 4 - **B** and Figure S13. This result also agrees with the potentiometric and spectrophotometric data, and it can be ascribed to the chemical exchange between the monoprotonated and the deprotonated forms of the complex (i.e., [PbHL]^−^ and [PbL]), which are concurrently present at the equilibrium between pH = 2 and pH = 4 (Figure 2). The p*K*_a_ of the deprotonation process ([PbHL]^+^ ⇋ [PbL] + H^+^) that can be estimated from the ^1^H NMR data (Figure S13: p*K*_a_ = 2.6 ± 0.1) is in good agreement with the value obtained by potentiometry (Table 1: 20.89–18.2 = 2.69).

As shown in Figure 4, the ^1^H NMR spectra of [Pb(DO4S)]^2+^ and [Pb(DO2A2S)] are characterized by a small number of almost exceptionally broad resonances, likely as a consequence of the high symmetry of the complexes combined with marked fluxional solution behavior. However, while the coordination of Pb^2+^ resulted in a significant local effect on the shifts and broadening of the resonances of the NCH_2_^1^H located on the macrocyclic ring and the pendant arms, it had a less marked influence on resonance broadening of the terminal sulfanyl protons (SCH_3_). This could suggest that the dynamic processes involve the macrocycle (e.g., ring turns) rather than the side chains.

**Figure 5:**
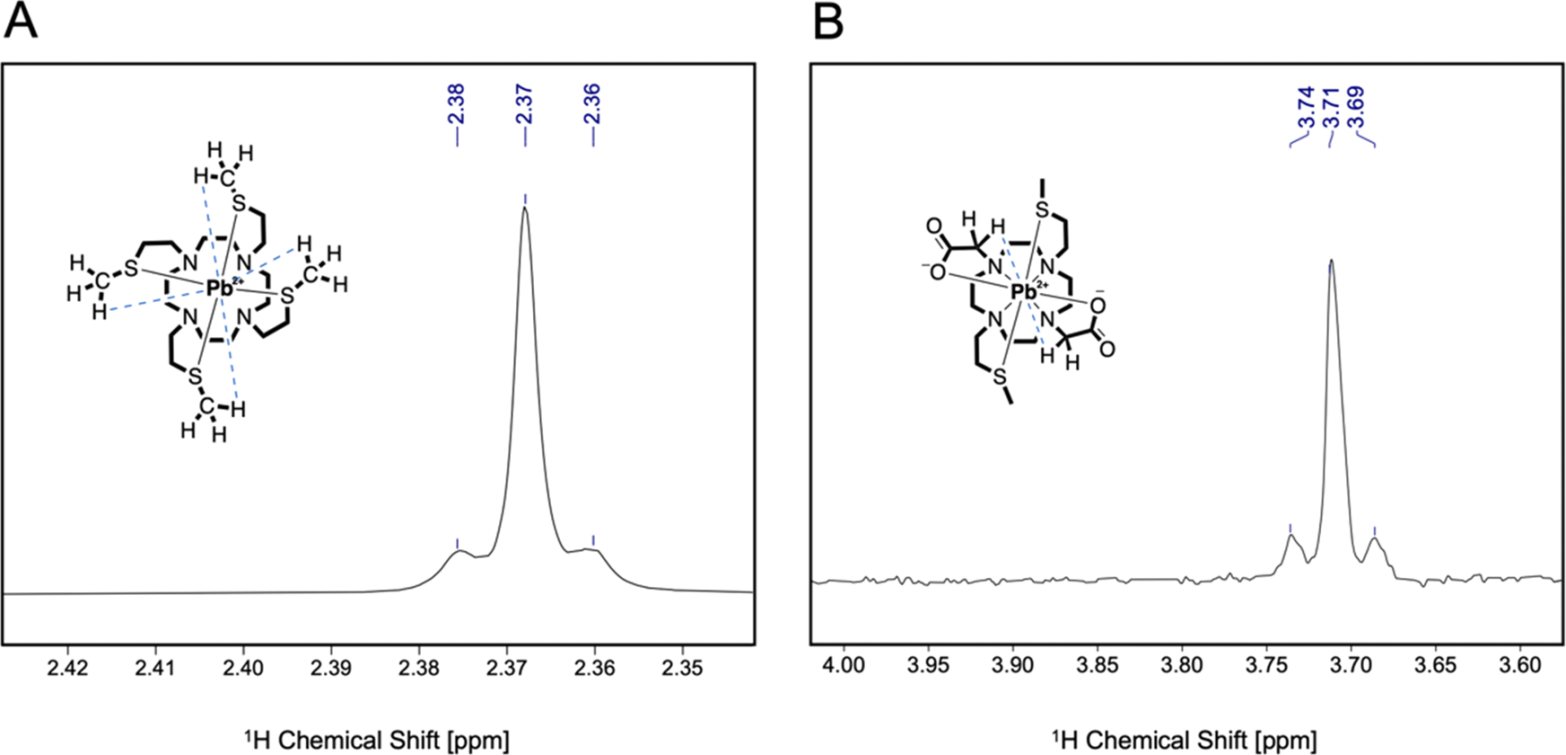
Satellites peaks of (A) [Pb(DO4S)]^2+^ and (B) [Pb(DO2A2S]. The ^1^H–^207^Pb interactions are outlined in blue.

The presence of a single resonance for the SCH_3_ and SCH_2_ and the acetate protons would indicate that they are either always bound to the metal center or in a rapid exchange on the NMR time scale. These two hypotheses can be evaluated by examining in more detail the SCH_3_ singlets. With regard to [Pb(DO4S)]^2+^ (Figure 4 - **A**), this signal (δ(SCH_3_) = 2.37 ppm) was accompanied by two satellite peaks at δ = 2.36 and 2.38 ppm (^3^*J* = 6.1 Hz, Figure 5 - **A**), which arise as a consequence of the ^1^H–^207^Pb coupling through the S atoms (Pb–S–CH_3_), with ^207^Pb being an NMR-active nucleus (spin = +1/2). The natural abundance of ^207^Pb (22.6%) nicely corresponded to the relative area of the satellite peaks (∼23%), thus indicating the simultaneous and static binding of all S atoms to the metal center. For [Pb(DO2A2S)], no satellites were present for the SCH_3_ protons (δ(SCH_3_) = 2.25 ppm) while for the CH_2_COOH signal (δ(CH_2_COOH) = 3.72 ppm) they were recognized (^3^*J* = 19 Hz, Figure 5 - **B**). These features suggest that the acetate arms are bound to Pb^2+^ in a nonfluxional mode while SCH_3_ exchanges faster on the NMR time scale. Alternatively, the absence of coupling between ^207^Pb and SCH_3_ protons in Pb^2+^-DO2A2S could be justified considering that the coupling constant is zero due to bond angle constraints.

The ^1^H NMR spectra of [Pb(DO3S)]^2+^ and [Pb(DO3SAm)]^2+^ exhibited more complicated patterns characterized by a larger number of sharper signals (Figure S7). The former feature likely arises from the intrinsic lower symmetry of the ligands themselves, while the latter could suggest a slowed-down fluxionality with respect to the more symmetric DO4S and DO2A2S. In [Pb(DO3S)]^2+^, the signals from chemically inequivalent N-1, N-7, and N-4 SCH_3_ protons resulted almost coincidently but still distinctly, thus indicating a slight asymmetry in the complex solution state (Figure S14 - A). The two satellite peaks at δ = 2.25 and 2.29 ppm confirm the ^1^H–^207^Pb coupling (^3^*J* = 15 Hz). It is worth noting that the coupling is only detectable for the SCH_3_ protons on N-1 and N-7 arms, thereby demonstrating that only the two symmetric S-donors are statically bound to Pb^2+^ with respect to the NMR time scale (Figure S14 - A). The asymmetric arm (N-4) may not be involved in the Pb^2+^ coordination or can be in a fast exchange, or the coupling could be absent for geometrical reasons which induce *J* = 0. However, since both the cyclen unit and the pendant resonances of [Pb(DO3S)]^2+^ are downfield shifted with respect to the uncoordinated ligand,^27^ it is reasonable to assume that each heteroatom interacts on average with Pb^2+^. In [Pb(DO3SAm)]^2+^, a slight inequivalence of the sulfanyl arms was evidenced by the presence of two almost overlapped singlets for the SCH_3_ protons (Figure S14 - B). The absence of any ^1^H–^207^Pb coupling pattern, combined with the deshielding of the resonances with respect to the unbound ligand, suggests that all of the S donors are bound to the Pb^2+^ center but in a rapid solution exchange.

**Figure 6:**
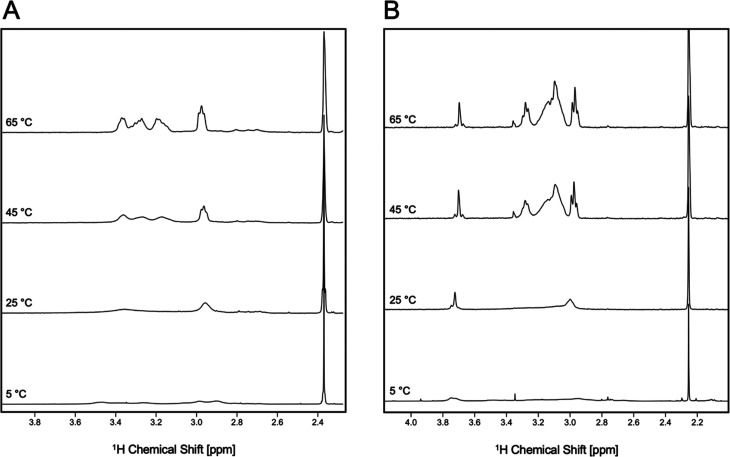
Variable-temperature ^1^H NMR spectra of (A) [Pb(DO4S)]^2+^ and (B) [Pb(DO2A2S)] (400 MHz, 90% H_2_O + 10% D_2_O, and *C*_Pb_^2+^ = *C*_ligand_ = 1.0 × 10^–3^ M).

To further probe the coordination structure of the Pb^2+^ complexes and their dynamic behavior, variable-temperature ^1^H NMR were collected. As reported in Figure 6 and in Table S4, signal sharpening was observed when acquiring the spectra at higher temperatures, from *T* = 5 °C to *T* = 65 °C, for [Pb(DO4S)]^2+^ and [Pb(DO2A2S)]^2+^. The broad signals observed at room temperature can thus be related to the fluxionality of the complexes in aqueous solution, which could include a decoordination–coordination flip of the side chains, macrocycle ring turns, and/or the formation of square antiprism (SAP)/twisted square antiprism (TSAP) isomers which are known to form when DOTA-like chelators bind heavy metal ions.^34^ The latter hypothesis was checked through DTF calculations, which allowed us to compute and compare the energies of these isomers: the results revealed a significant energy difference between the SAP and TSAP forms (ΔΔ*G*_SAP–TSAP_ = 6.9 kcal/mol, in water). This suggests that the coexistence of these two isomers in solution is unlikely, emphasizing the thermodynamic dominance of the TSAP form over its counterpart at all investigated temperatures. As the NCH_2_ ring signals resemble the simpler spectra of [Pb(cyclen)]^2+^, where the multiplets arose from neighboring protons on the macrocyclic scaffold, becoming diasterotopic upon Pb^2+^ coordination and coupling to each other, the involvement of all N in the metal coordination sphere is supported.^35^ The SCH_2_ proton also exhibits temperature-dependent variations, unlike the SCH_3_ protons, which consistently appear as singlets. A slight peak broadening combined with the loss of the satellite peaks was observed at *T* > 45 °C for [Pb(DO4S)]^2+^. This could suggest that at higher temperatures the sulfanyl side chains became chemically equivalent through fast intramolecular exchange with respect to the NMR time scale.

When Pb^2+^ is bound to DO2A2S, the ^1^H–^207^Pb satellite peaks related to the acetate groups were present at *T* ≥ 25 °C, indicating that these donors are all bound to Pb^2+^. At *T* < 25 °C, this signal became broader and started to split: a slower transient dissociation and recoordination of O donors can therefore be deduced (Figure 6 - **B**).

[Pb(DO3S)]^2+^ and [Pb(DO3SAm)]^2+^ did not show significant temperature-dependent variation of their resonances. This further suggests a more rigid coordination environment for these complexes than for [Pb(DO4S)]^2+^ and [Pb(DO2A2S)]. The solution fluxionality seems therefore to be correlated with the symmetry of the ligands, as DO4S and DO2A2S are highly symmetric whereas DO3S and DO3SAm are not.

**Figure 7:**
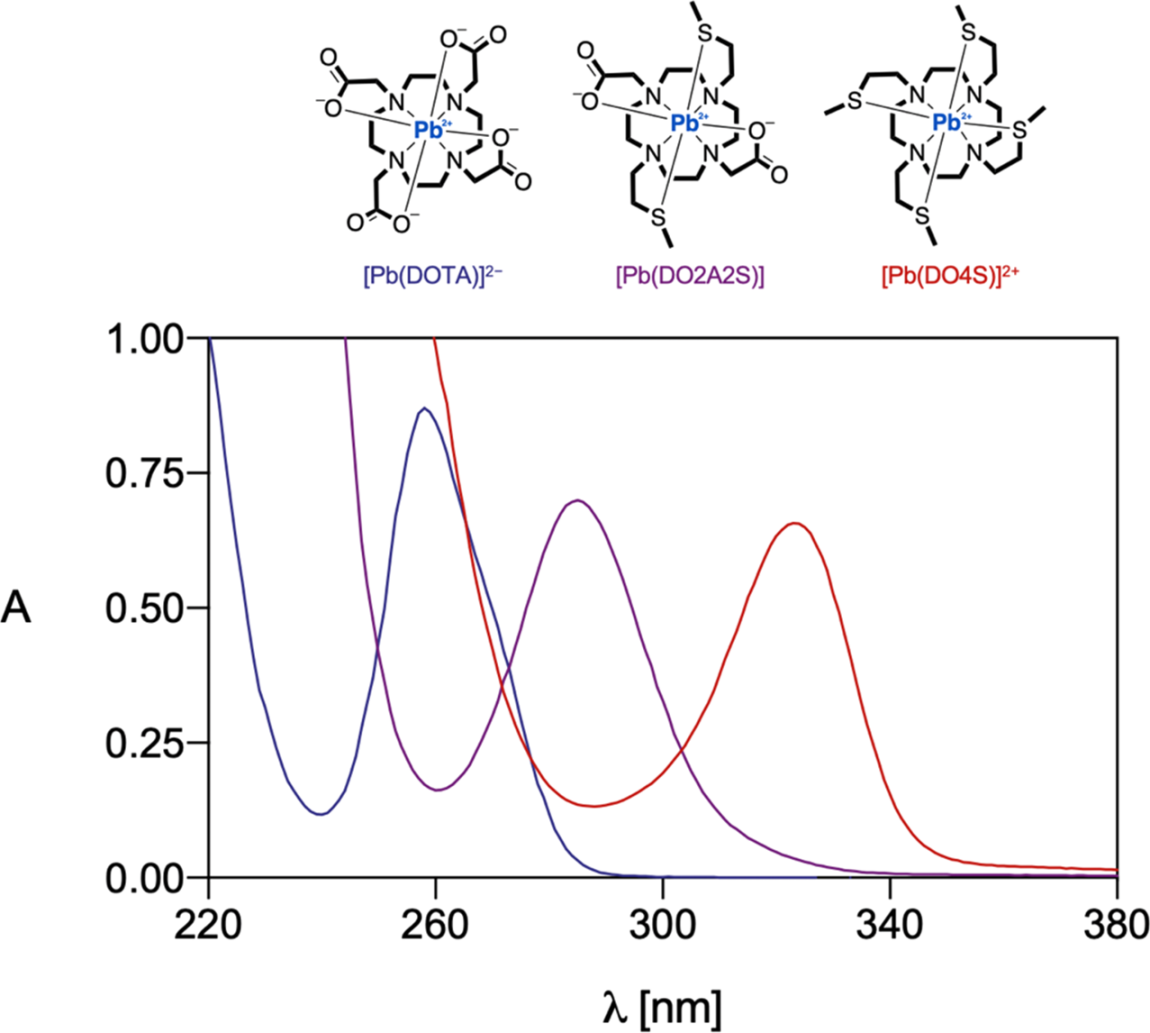
Comparison of the electronic spectra of [Pb(DOTA)]^2+^, [Pb(DO2A2S)], and [Pb(DO4S)]^2–^. Data from the former were taken from the literature.^32^

The role of the sulfanyl arms in the Pb^2+^ coordination is also indicated by the observed UV-Vis absorption maxima shift toward higher wavelengths, which occurred in the electronic spectra of the Pb^2+^ complexes when the set of S-donor atoms increased from DOTA to DO4S (Figure 7). The absorption band, attributed to the 6*s*^2^ to 6*sp* transition of the metallic center, experienced a shift that resembled what happened on substituting water ligands on the Pb^2+^ ion with more covalently binding donors as reported in the literature for different ligands.^36^ Consequently, the electronic transition shift to lower energy, due to the increasing covalency of the Pb–L bond, can be justified if the involvement of more covalently binding S donors in the Pb^2+^ coordination sphere is considered.

### 2.4 Acid-Mediated Dissociation Kinetics of Pb^2+^ Complexes

Although thermodynamic parameters are very valuable as a first gauge to assess the performance of a chelator for nuclear medicine applications, kinetic factors may become prevalent and determine the *in vivo* integrity of the resulting complex.^37^ Therefore, to complement the equilibrium studies, the kinetic inertness of the Pb^2+^ complexes was assessed by investigating their dissociation in acidic media (HCl) at room temperature by UV–Vis spectroscopy. Despite the fact that these conditions do not have a physiological equivalent, the study aims to evaluate the behavior of the complex under highly competitive and stressful conditions where their integrity can be compromised.

Representative spectral changes during the acid decomplexation assays are reported in Figures S15–S17. The observed dissociation rates (^d^*k*_obs_) and the corresponding half-life (*t*_1/2_) are collected in Table 3.

**Table 3:** ^d^*k*_obs_ and Half-Life (*t*_1/2_) for the Acid-Assisted Dissociation Reactions of the Pb^2+^ Complexes with DO4S, DO3S, DO3SAm, and DO2A2S in Aqueous HCl at Room Temperature

	^**d**^***k***_**obs**_ **[min**^**–1**^**]**			
***C***_**HCl**_ **[M]**	**[Pb(DO4S)]**^**2+**^	**[Pb(DO3S)]**^**2+**^	**[Pb(DO3SAm)]**^**2+**^	**[Pb(DO2A2S)]**
0.01	(29 ± 3) 10^–4^	(6 ± 1) 10^–2^	(3.0 ± 0.2) 10^–2^	(4.6 ± 1.3) 10^–2^^a^
0.1	(3.8 ± 0.7) 10^–2^	1.5 ± 0.3	0.43 ± 0.02	0.7 ± 0.3
1.0	1.04 ± 0.05	b	b	b

***t***_**1/2**_ **[min]**				
***C***_**HCl**_ **[M]**	**[Pb(DO4S)]**^**2+**^	**[Pb(DO3S)]**^**2+**^	**[Pb(DO3SAm)]**^**2+**^	**[Pb(DO2A2S)]**
0.01	240 ± 25	12 ± 2	22 ± 4	15 ± 4^a^
0.1	18 ± 3	0.47 ± 0.09	1.6 ± 0.2	1.1 ± 0.4
1.0	0.66 ± 0.03	b	b	b

a Not quantitative decomplexation.

b Instantaneous decomplexation during the reagent mixing time.

The Pb^2+^ complexes formed by DO2A2S partially decomplexed at pH = 2, and the decomplexation extent increased at higher proton concentration. [Pb(DO4S)]^2+^, [Pb^2+^(DO3S)]^2+^, and [Pb(DO3SAm)]^2+^ decomplexed to a larger extent. These results agree with the thermodynamic data reported in Figure 2.

[Pb(DO4S)]^2+^ was demonstrated to be the most inert complex with respect to the acid-mediated dissociation along the series of investigated ligands (Table 3). The inertness of the other complexes was fairly similar and lower than that of [Pb(DO4S)]^2+^. While the presence of O donors in the pendant arms of DO2A2S and DO3SAm increased the complexes’ stability (*vide supra*), the greatest number of S donors in DO4S increased its inertness likely because no acid-base competitive equilibria occur on the SCH_3_ binding moiety. On the other hand, the comparable inertness of [Pb(DO3S)]^2+^ with respect to the carboxylic/amide derivatives could be related to the nonfully saturated coordination sphere around the metal center that can generate a labile site.

If the dissociation parameters are compared with the literature values for Pb^2+^-DOTA, then it is evident that the sulfur-bearing Pb^2+^ complexes are more labile under acidic conditions compared to the former (e.g., *t*_1/2_ [0.1 M] = 33 min, *t*_1/2_ [1 M] = 3.8 min for Pb^2+^-DOTA). This could be related to the higher thermodynamic stability of the Pb^2+^-DOTA complex.^32^

### 2.5 Radiolabeling with [^203^Pb]Pb^2+^

**Figure 8:**
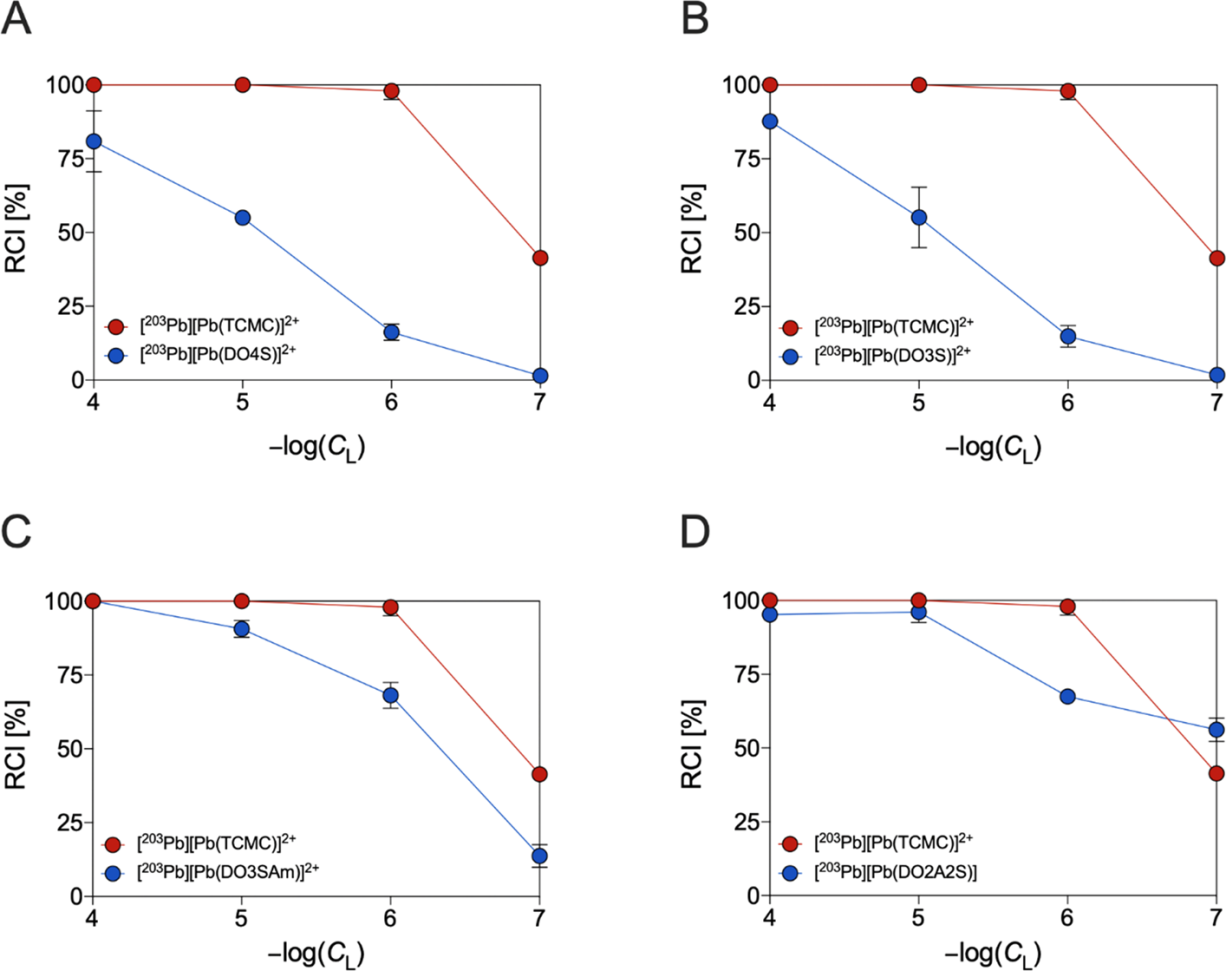
Comparison of the [^203^Pb]Pb^2+^ incorporation yields at different ligand concentrations for the investigated S-bearing cyclen-based macrocycles and the state-of-the-art TCMC at pH = 7 and room temperature (1 h of reaction time).

Concentration-dependent radiolabeling studies with [^203^Pb]Pb^2+^ were conducted under mild reaction conditions (room temperature, pH = 7) to determine the ability of the S-bearing ligands to coordinate Pb^2+^ in extremely low concentrations. The obtained results are shown in Figure 8.

The state-of-the-art chelator for Pb^2+^ complexation (i.e., TCMC) gave quantitative radiochemical incorporation (RCI) at ligand concentrations higher than 10^–6^ M while the RCI dropped to 41 ± 1%, lowering its concentration to 10^–7^ M. When the amide donors of TCMC were swapped with sulfanyl arms in DO4S and DO3S, the RCI reduced sequentially from 81 ± 10% at 10^–4^ M to 1.4 ± 0.4% at 10^–7^ M for the former and from 88 ± 1% at 10^–4^ M to 2 ± 1% at 10^–7^ M for the latter. The presence of carboxylic/amide donors in DO2A2S and DO3SAm drastically improved the incorporation yields when compared with the pure S-bearing analogues, in complete agreement with our thermodynamic results (Table 1). Indeed, both DO2A2S and DO3SAm were able to efficiently complex [^203^Pb]Pb^2+^ at ligand concentrations of 10^–5^ M and 10^–4^ M (RCIs > 90%). At 10^–6^ and 10^–7^ M, the RCI decreased to 68 ± 1 and 56 ± 4% for the former and to 68 ± 4 and 14 ± 4% for the latter. DOTA was previously found to be able to complex [^203^Pb]Pb^2+^ with RCIs of 96 ± 1, 76 ± 9, 3 ± 1, and 1.5 ± 0.2% at 10^–4^, 10^–5^, 10^–6^, and 10^–7^ M, respectively.^11^ The obtained results pointed out that, under these conditions, DO2A2S and DO3SAm are superior to DOTA and slightly less efficient than TCMC at complexing [^203^Pb]Pb^2+^.

As shown in Figure S18, a change in the reaction times had no effect on RCI for DO2A2S and DO3SAm or for TCMC. On the other hand, DO4S and DO3S demonstrated better reactivity by increasing the time, as the RCIs increased from 35% after 5 min to 81% after 1 h for the former and from 65% after 5 min to 88% after 1 h for the latter. The reactivity trend observed during the [^203^Pb]Pb^2+^ labeling experiments reflects the results obtained during the kinetic evaluation with stable Pb^2+^ (*vide supra*).

Radiolabeling studies of TACD3S, TRI4S, and TE4S were conducted as a benchmark to further probe the instability of their Pb^2+^ complexes (*vide supra*). They revealed a poor ability to coordinate [^203^Pb]Pb^2+^, as no radiometal incorporation was observed at room temperature albeit using the highest ligand concentration assessed (10^–4^ M). RCIs were only vaguely improved through heating at *T* = 80 °C (Figure S19).

### 2.6 Human Serum Integrity of [^203^Pb]Pb^2+^ Complexes

**Figure 9:**
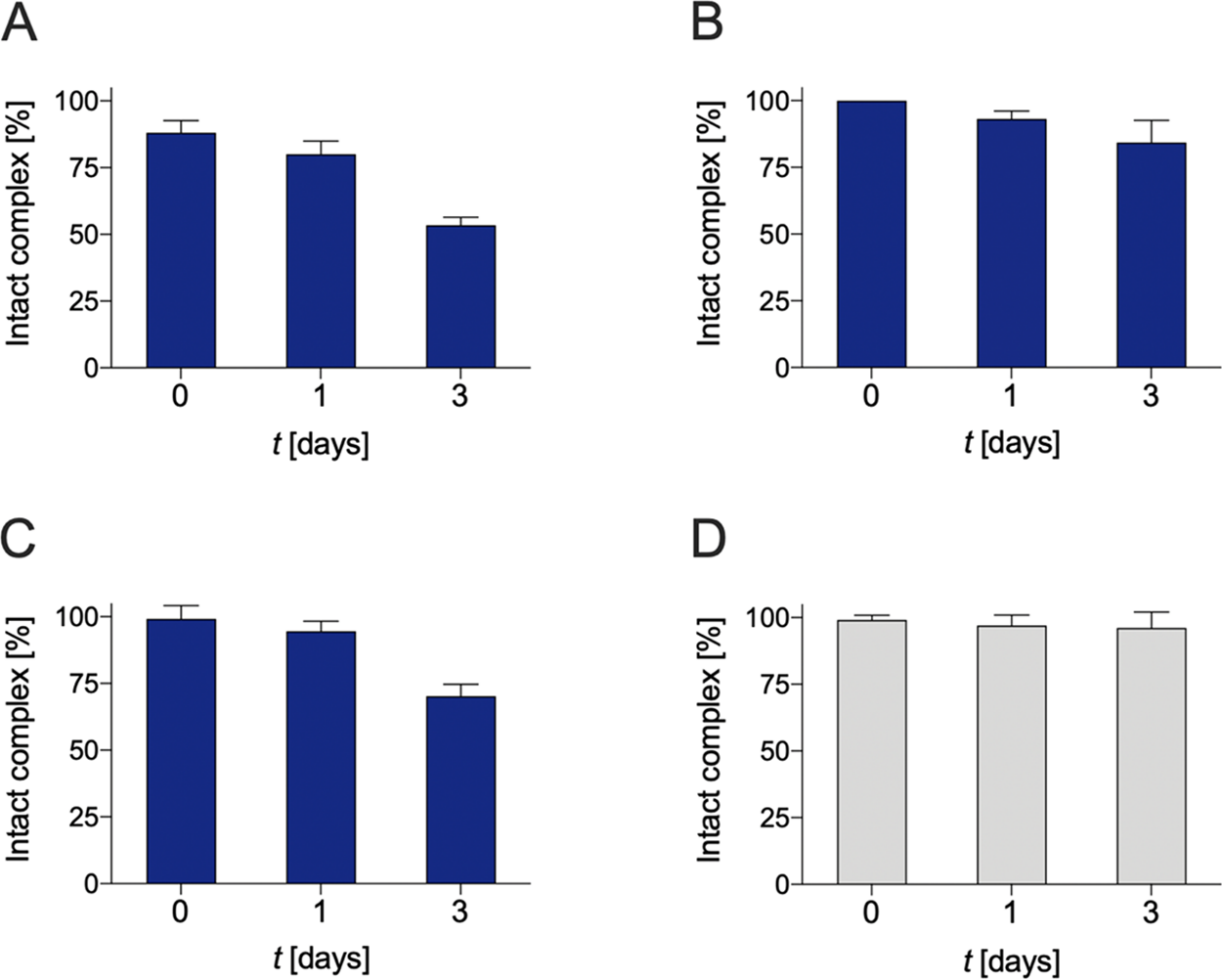
Integrity of (A) [^203^Pb][Pb(DO4S)]^2+^, (B) [^203^Pb][Pb(DO3SAm)]^2+^, (C) [^203^Pb][Pb(DO2A2S)], and (D) [^203^Pb][Pb(TCMC)]^2+^ in human serum at 37 °C over 3 days.

The human serum integrity of [^203^Pb][Pb(DO4S)]^2+^, [^203^Pb][Pb(DO3SAm)]^2+^, and [^203^Pb][Pb(DO2A2S)] was evaluated to assess their robustness in the presence of biologically relevant substrates that can compete and displace the chelator-bound metal ions *in vivo*. The obtained results are displayed in Figure 9.

[^203^Pb][Pb(DO4S)]^2+^ was appreciably stable in human serum after 24 h (80 ± 5% was detected as an intact complex), while a significant decomplexation was observed over 3 days (53 ± 3%). [^203^Pb][Pb(DO3SAm)]^2+^ and [^203^Pb][Pb(DO2A2S)] retained markedly high integrity over the course of 24 h, with <5% transchelation (likely for serum proteins). Only at very long time points (i.e., after 3 days) a progressive decrease in the percentage of the intact complex was observed (84 ± 8% for [^203^Pb][Pb(DO3SAm)]^2+^ and 70 ± 4% for [^203^Pb][Pb(DO2A2S)]). In comparison, [^203^Pb][Pb(TCMC)]^2+^ was found to be more robust.

## 3 Experimental Section

### 3.1 Materials and Methods

All chemicals were obtained from commercial suppliers and were used as received without further purification. 1,4,7,10-tetraaza-1,4,7,10-tetra-(2-carbamoylmethyl)-cyclododecane (TCMC) was purchased from Chematech. DO4S, DO3S, DO3SAm, DO2A2S, TACD3S, TRI4S, and TE4S were synthesized in our laboratories according to the literature procedures.^27,28^ All solutions were prepared using ultrapure water (18.2 MΩ/cm) purified with a Purelab Chorus (Veolia) or a Milli-Q Millipore system.

### 3.2 Formation Kinetics

The formation kinetics of the Pb^2+^ complexes was evaluated at room temperature by UV–Vis or ^1^H NMR spectroscopy. A typical experiment was performed by mixing equimolar amounts of metal and ligand solutions (final concentrations: 1.0 × 10^–4^ M for UV–Vis, 1.0 × 10^–3^ M for ^1^H NMR) in buffered media at pH = 2 (HCl 10^–2^ M), pH = 3.7 (formic/formiate buffer), pH = 5 (acetic/acetate buffer), and pH = 7.4 (2-[4-(2-hydroxyethyl)piperazin-1-yl]ethanesulfonic acid, i.e., HEPES, buffer). To avoid the formation of PbCO_3_ (*K*_s_ = 7.4 × 10^–14^), water was boiled before each measurement.

The electronic spectra were collected in the 200–800 nm range at different time points using a Cary 60 UV–Visible spectrophotometer (Agilent) equipped with a 1 cm path length quartz cell. The complexation reactions were monitored by the increase in the absorption peaks diagnostic of the Pb^2+^-ligand complex formation at the characteristic wavelength (Table S2) over time. ^1^H NMR spectra were recorded on a 400 MHz Bruker Avance III HD spectrometer. All data were collected and processed with Topspin 3.5 using standard Bruker processing parameters with Topspin 4.1.1 software. Chemical shifts (δ) are reported as parts per million (ppm) and are referenced to trimethylsilyl propionic acid sodium salt (TSP) (Sigma-Aldrich, 99%). Water suppression was carried out with the excitation sculpting pulse scheme.^38^

### 3.3 Thermodynamic Measurements

The experimental procedures and details of the apparatus followed our previously reported studies.^2,4,28^ Herein a brief description is given.

#### 3.3.1 Solution Preparations

Nitric acid (HNO_3_) solutions were prepared at ∼10^–1^ M by dilution of the concentrated acid (Aristar - VWR Chemicals, 69%) and standardized against sodium carbonate (Aldrich, 99.95–100.5%). Carbonate-free sodium hydroxide (NaOH) solutions were prepared at ∼10^–1^ M from commercial pellets (Fluka, 99% min) using freshly boiled ultrapure water and standardized using the previously standardized acid. The ligand stock solutions were prepared by direct dissolution of a weighed portion of ligand in water at ∼10^–3^ M. HNO_3_ was coadded to increase the solubility and avoid carbonatation phenomena. Pb^2+^ solutions were preprepared from analytical-grade nitrate salt (Pb(NO_3_)_2_, Sigma-Aldrich, 99%) and standardized using complexometric titrations with ethylenediaminetetraacetic acid (EDTA) with xylenole orange as an indicator. The ionic strength (*I*) was fixed to 0.15 M using sodium nitrate (NaNO_3_) as background electrolytes. All of the experiments described below were repeated at least in triplicate.

#### 3.3.2 Potentiometric Titrations

Direct (in-cell) pH-potentiometric titrations were carried out with an automated Metrohm 765 Dosimat titrating system using a Metrohm 713 pH meter. A Hamilton pH 0–14 glass electrode was employed which was daily calibrated by direct titration of HNO_3_. Ligand (DO2A2S) and Pb^2+^ were introduced in a 3 mL glass titration cell thermostated at 25 ± 1 °C with a Haake F3 cryostat in concentrations ranging from 0.85 to 2.0 × 10^–3^ M. The metal-to-ligand molar ratio varied from 0.5:1 to 2:1. The solutions were acidified with a known volume of HNO_3_ to adjust the starting pH to 2, and the titrations were then carried out by the addition of known volumes of NaOH stock solution over the pH range of 2–12. Before and during all of the titrations, a constant flow of N_2_ was maintained over the sample solution to remove CO_2_.

#### 3.3.3 UV–Vis Titrations

UV–Vis spectrophotometric titrations were carried out by the out-of-cell method in the pH range of 0–12 at *T* = 25 °C. Stock solution of the ligands (10^–3^ M) and Pb(NO_3_)_2_ (10^–2^ M) were mixed in separated vials in a 1:1 metal to ligand molar ratio (final concentrations: *C*_Pb^2+^_ = *C*_ligand_ = 1 × 10^–4^ M), and different amounts of previously standardized HNO_3_ and/or NaOH were added to adjust the pH. A Mettler Toledo pH meter with a glass electrode was used to measure the pH. The latter was daily calibrated using commercial buffer solutions (pH = 4.01 and 7.01 at *T* = 25 °C). In highly acidic solutions (pH ≪ 2), the pH was computed from the acid concentration (pH = −log*C*_H^+^_). After the pH adjustment, the vials were sealed, heated to *T* = 60 °C to ensure complete Pb^2+^ complexation, and cooled to room temperature. The absorption spectra were recorded using the same apparatus described for the kinetic measurements (*vide supra*). The equilibrium was reached when no variations of the electronica spectra or the pH were detected.

#### 3.3.4 NMR

##### 3.3.4.1 pH-Dependent ^1^H NMR Titrations

^1^H NMR spectra of the Pb^2+^ complexes were recorded at different pH. All of the solutions were prepared in 90% H_2_O + 10% D_2_O (Sigma-Aldrich, 99.9% D) at ∼10^–3^ M concentration, and the pH was adjusted with small additions of HNO_3_ and/or NaOH and measured with the same setup used for the UV–Vis spectrophotometric measurements.

##### 3.3.4.2 Variable-Temperature ^1^H NMR

The spectra of the Pb^2+^ complexes were recorded at different temperatures using a 400 MHz Bruker Avance III HD spectrometer in the temperature range of 5–65 °C.

#### 3.3.5 Data Treatment

All equilibrium data were processed using the least-squares fitting program PITMAP as described in our previous works.^2,4,28^ The overall equilibrium constants refer to the general equilibria *p*M^*m*+^ + *q*H^+^ + *r*L^*l*–^ ⇆ M_*p*_H_*q*_L_*r*_^*pm+q–rl*^, where M^*m*+^ represent the metal ion and L^*l*–^ represents the deprotonated ligand, and are designated as logβ (β_*pqr*_ = [M_*p*_L_*q*_H_*r*_]/[M]^*p*^[L]^*q*^[H]^r^). The refinements of log β included the previously determined ligand protonation constants and the Pb^2+^ hydrolysis products whose equilibrium constants were fixed to the literature values.^27,39^ Each set of data was treated independently and then merged and treated simultaneously to give the final stability constants. The errors quoted are the standard deviations of the overall stability constants calculated by the PITMAP program.

### 3.4 DFT Calculations

All calculations were performed using the Amsterdam density functional (ADF) software package.^40−42^ The GGA exchange-correlation functional OPBE was used for the optimization of all stationary points, in combination with the TZP (triple-ζ quality augmented with one set of polarization functions on each atom) basis set.^43−45^ Frequency calculations were subsequently carried out at the same level of theory to assess the nature of the stationary points (zero imaginary frequencies were computed, denoting that the geometries correspond to minimum-energy structures on the potential energy surface - PES). A more accurate energy evaluation has been carried out for the optimized geometries using the same exchange-correlation potential combined with the TZ2P basis set (triple-ζ quality augmented with two sets of polarization functions on each atom). Scalar relativistic effects were accounted for using the zeroth-order regular approximation (ZORA).^46^ This level of theory is denoted in the text as ZORA-OPBE/TZ2P//ZORA-OPBE/TZP, and it has been adopted successfully for the description of molecular systems containing heavy nuclei.^4,27,47−49^ All of the calculations were performed in the gas phase and in water; for the latter case, the solvation effects have been treated using the COSMO (COnductor-like Screening MOdel) approach (level of theory: COSMO-ZORA-OPBE/TZ2P//ZORA-OPBE/TZP). A radius of 1.93 Å and a relative dielectric constant of 78.39 were used. The empirical parameter in the COSMO equation was considered to be 0.0. The radii of the atoms are the classical MM3 radii divided by 1.2.

### 3.5 Acid-Mediated Dissociation Kinetics

The dissociation kinetics of the Pb^2+^ complexes were studied under pseudo-first-order conditions at room temperature without control of the ionic strength by the addition of a concentrated aqueous solution of HCl (0.01 to 1 M) to aqueous solutions of the preformed complexes. The concentration of the Pb^2+^ complexes after the H^+^ addition was 1.0 × 10^–4^ M. The dissociation reaction was followed by the decreasing intensity of the absorption band of the complexes at the characteristic wavelength (Table S2) using the same apparatus described for the formation kinetic measurements (*vide supra*).

The data were processed and fitted using the equation ln*A*_t_ = ln*A*_0_ – ^d^*k*_obs_*t*, where *A*_t_ and *A*_0_ are the absorbances at time *t* and at the beginning of the reaction and ^d^*k*_obs_ is the observed dissociation rate constant. The corresponding half-life was obtained from the equation *t*_1/2_ = ln(2)/^d^*k*_obs_.

### 3.6 [^203^Pb]Pb^2+^ Radiolabeling

#### 3.6.1 Production

[^203^Pb]Pb^2+^ was produced via the ^203^Tl (p,n)^203^Pb reaction at TRIUMF’s TR13 cyclotron following a previously reported method.^11^ [^203^Pb]Pb^2+^ was obtained as [^203^Pb]Pb(OAc)_2_ in ammonium acetate (1 M, pH = 7) solution.

#### 3.6.2 Radiolabeling

Stock solutions of the ligands (10^–3^ M) were prepared in ultrapure deionized H_2_O and diluted appropriately to give a serial dilution series (10^–4^–10^–6^ M). Concentration-dependent radiolabeling was performed by the addition of [^203^Pb]Pb^2+^ (10 μL, 124 kBq) to a solution containing the ligand (10 μL, 10^–3^–10^–6^ M) diluted in NH_4_OAc buffer (80 μL, 1 M, pH = 7). Water replaced the ligands in the negative control. All of the radiolabeling for the cyclen-based derivatives was performed at room temperature and monitored at 5 min and 1 h time points whereas heating at *T* = 80 °C was also employed for TACD3S, TRI4S, and TE4S. All radiolabeling reactions were repeated at least in triplicate.

Radiochemical incorporation (RCI) was determined via instant thin-layer chromatography (iTLC) with silicic acid (SA)-impregnated paper TLC plates (iTLC-SA, Agilent Technologies, USA). Ethylenediamine tetraacetic acid (EDTA, 50 mM, pH = 5.5) was used as the eluent. Under these conditions, free [^203^Pb]Pb^2+^ migrates with the solvent front (*R*_f_ = 1) while the [^203^Pb]Pb^2+^ complexes remain at the baseline (*R*_f_ = 0). The iTLC plates were analyzed on an Eckert & Ziegler AR-2000 TLC scanner, and all the data were processed with Eckert & Ziegler WinScan software. Representative TLC radiochromatograms are presented in Figure S20.

### 3.7 Human Serum Integrity

The integrity of the [^203^Pb]Pb^2+^ complexes, prepared using the radiolabeling protocol described above, was assessed by incubation in human serum at *T* = 37 °C (1:1 *V*/*V* dilution) at varying time points. The metal-complex stability was monitored over the course of 3 days via iTLC using the same protocol described for the radiolabeling studies.

## 4 Conclusions

A series of polyazamacrocyclic ligands incorporating S-donor pendants were investigated as potential ligands for the chelation of the [^203/212^Pb]Pb^2+^ theranostic pair. The rationale behind the selection of the investigated chelators was the hypothesis that the introduction of sulfanyl pendants could improve the stability and the inertness of the resulting Pb^2+^ complexes over their carboxylic acid/amide-bearing counterparts, as softer donors could optimally complement the borderline-soft nature of Pb^2+^.

Contrary to our expectations, UV–Vis spectrophotometric, NMR, and pH-potentiometric titrations, combined with DFT calculations, revealed that the introduction of S-donors on the arms appended on the cyclen scaffold induced a progressive drop in the thermodynamic stability of the resulting Pb^2+^ complexes, by about 5 log units from DO2A2S (pPb = 15.7) to DO4S (pPb = 10.2). On the other hand, the greatest number of S donors in DO4S increased its kinetic inertness in highly acidic environments with respect to the O-containing analogues, DO2A2S and DO3SAm.

NMR studies gave insight into the geometry of the Pb^2+^ complexes, giving evidence that S was involved in the metal coordination. An average highly fluxional and symmetric complex was formed in solution when Pb^2+^ was bound to DO4S or DO2A2S, whereas the introduction of asymmetry into the ligand structure in DO3S and DO3SAm, albeit maintaining unvaried the concurrent role of all of the donors in the metal binding, afforded a more static coordination environment.

No complexation was observed when the cyclen core was substituted with different macrocyclic rings in TACD3S, TRI4S, and TE4S, likely a result of a mismatch between the metal ion and the ring cavity. This outcome highlights the importance of considering the correct macrocyclic platform for the future development of tailored macrocyclic chelators for [^203/212^Pb]Pb^2+^.

To evaluate the complexation efficiency of the cyclen-based ligands under extremely dilute conditions, concentration-dependent radiolabeling with [^203^Pb]Pb^2+^ was performed. While DO4S and DO3S displayed modest labeling performances, DO2A2S and DO3SAm demonstrated quantitative radiochemical incorporation under mild conditions (room temperature, 5 min reaction time) at a chelator concentration of as low as 10^–5^ M.

As a final assessment of the potential of these architectures for [^203/212^Pb]Pb^2+^ chelation, the human serum integrity of the corresponding [^203^Pb]Pb^2+^ complexes was evaluated. [^203^Pb][Pb(DO4S)]^2+^ was only moderately inert (80 ± 5%) whereas [^203^Pb][Pb(DO3SAm)]^2+^ (93 ± 1%) and [^203^Pb][Pb(DO2A2S)] (94 ± 1%) demonstrated an encouraging robustness at least over 24 h. The ability to form an intact complex in human serum makes DO3SAm and DO2A2S viable candidates for further diagnostic and therapeutic applications with [^203/212^Pb]Pb^2+^ once coupled to a tumor-seeking moiety.

## Supporting Information Available

UV–Vis and ^1^H NMR spectra (mono- and bidimensional) of the solutions containing each ligand and Pb^2+^ at various pH and time points (tables and figures), timing required to reach equilibrium in metal + ligand solutions (table), radiochemical incorporation of [^203^Pb]Pb^2+^, representative iTLC radio-chromatograms (figures), and references (PDF)
