# Supplementary material for: Tuning the Softness of the Pendant Arms and the Polyazamacrocyclic Backbone to Chelate the 203Pb/212Pb Theranostic Pair
Source: Inorg Chem. 2024 Jan 17;63(4):1745–58. doi: 10.1021/acs.inorgchem.3c02610 (PMC10828988; doi:10.1021/acs.inorgchem.3c02610)
Supplement: Supplementary file 1 — ic3c02610_si_001.pdf [file ic3c02610_si_001.pdf]

Supplementary Information

Tuning the Softness of the Pendant Arms and the Polyazamacrocyclic Backbone to Chelate the $^{203}Pb/^{212}Pb$ Theranostic Pair

Marianna Tosato[1,2,3,4], Parmissa Randhawa[3,4], Luca Lazzari[1], Brooke L. McNeil[3,4], Marco Dalla Tiezza[1], Giordano Zanoni[1], Fabrizio Mancin[1], Laura Orian[1], Caterina F. Ramogida[3,4], Valerio Di Marco[1,*]

[1] Department of Chemical Sciences, University of Padova, 35131 Padova, Italy

[2] Radiopharmaceutical Chemistry Section, Nuclear Medicine Unit, AUSL-IRCCS Reggio Emilia, 42122 Reggio Emilia, Italy

[3] Department of Chemistry, Simon Fraser University, Burnaby, British Columbia, V5A 1S6, Canada

[4] Life Sciences Division, TRIUMF, Vancouver, British Columbia, V6 T2A3, Canada

* **Corresponding Author**: valerio.dimarco@unipd.it

Supplementary Figures

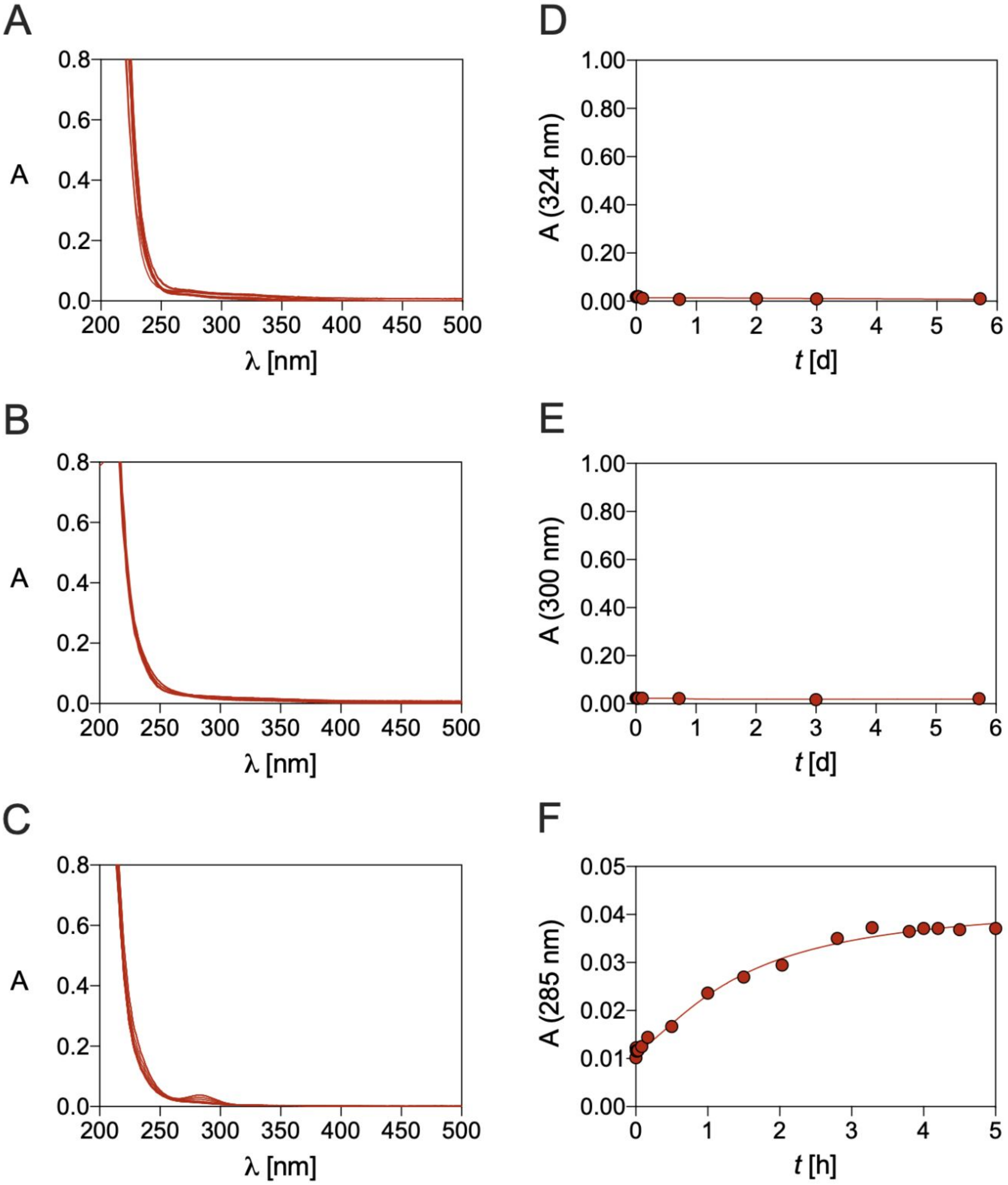

Figure S1. Representative UV-Vis spectra related to the formation kinetics of the Pb^{2+} complexes at pH 2 with (A) DO4S, (B) DO3S and (C) DO2A2S ($C_{Pb^{2+}}$ = C_{ligand} = 1.0·10^{-4} M). (D, E, F) Variation of $A_{\lambda max}$ *vs.* time and trend line for DO4S and DO3S. No spectral variations were observed even after prolonged heating.

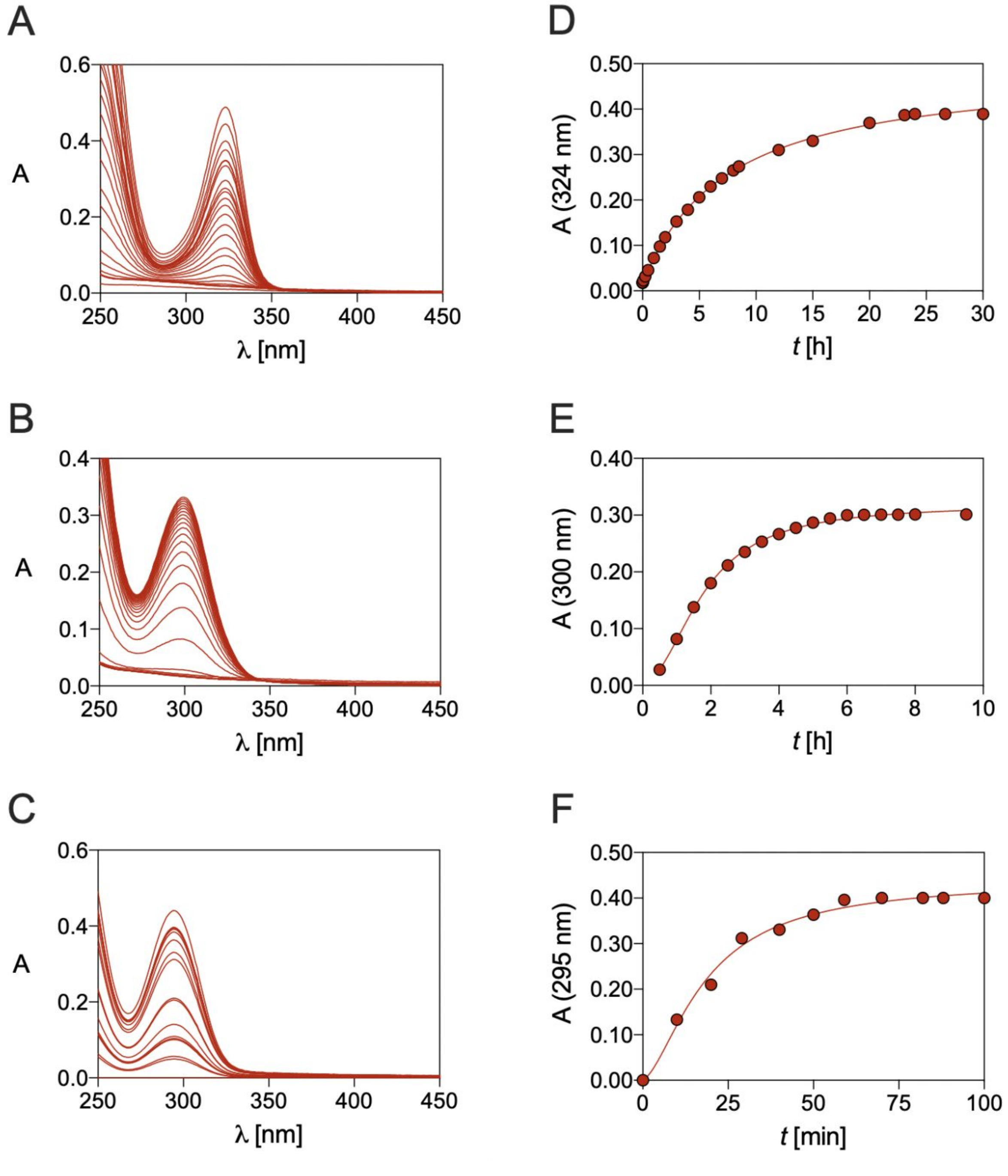

Figure S2. Representative UV-Vis spectra related to the formation kinetics at pH 5 of the Pb^{2+} complexes with (A) DO4S, (B) DO3S and (C) DO3SAm ($C_{Pb^{2+}} = C_{ligand} = 1.0 \cdot 10^{-4}$ M). (D, E, F) Variation of $A_{\lambda max}$ *vs.* time and trend line.

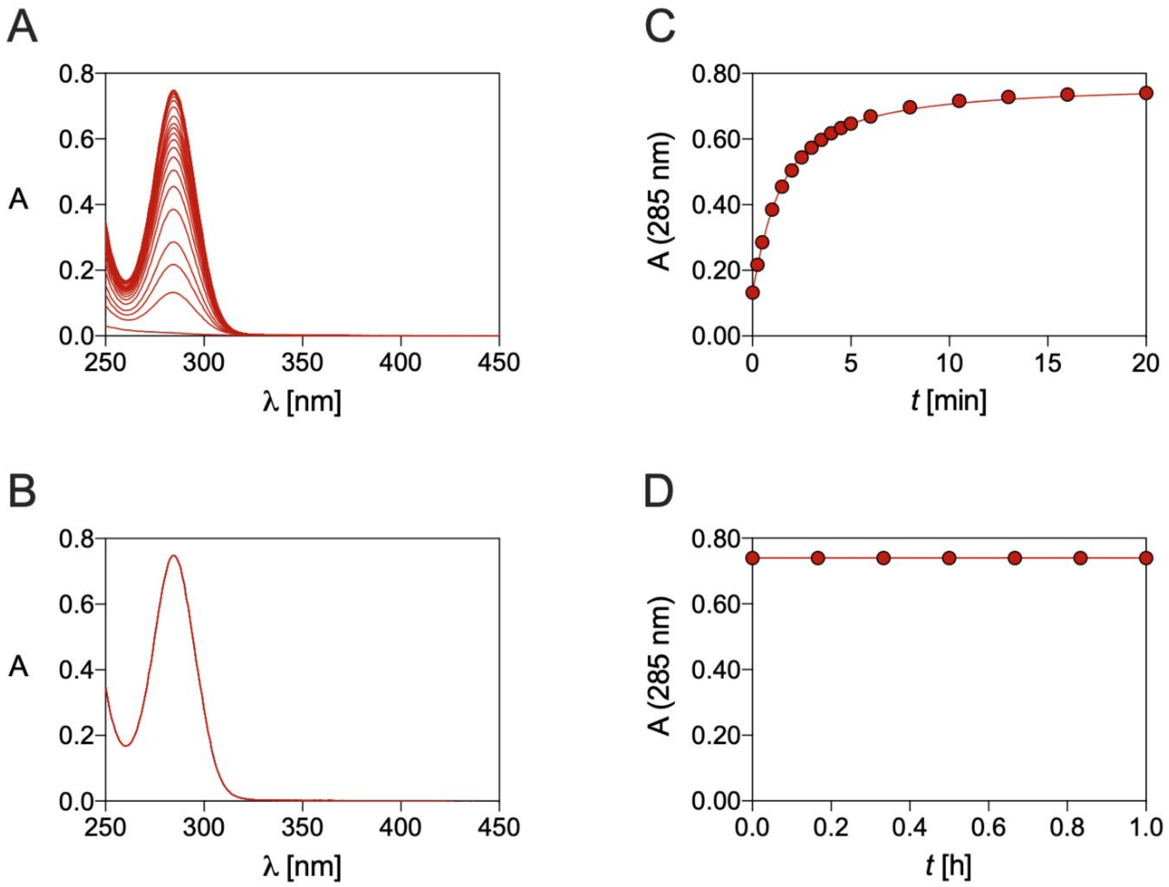

Figure S3. Representative UV-Vis spectra related to the formation kinetics at (A) pH 3.7 and (B) pH 5 of the Pb^{2+} complexes with DO2A2S ($C_{Pb^{2+}} = C_{DO2A2S} = 1.0 \cdot 10^{-4}$ M). (C, D) Variation of $A_{\lambda,max}$ *vs.* time and trend line.

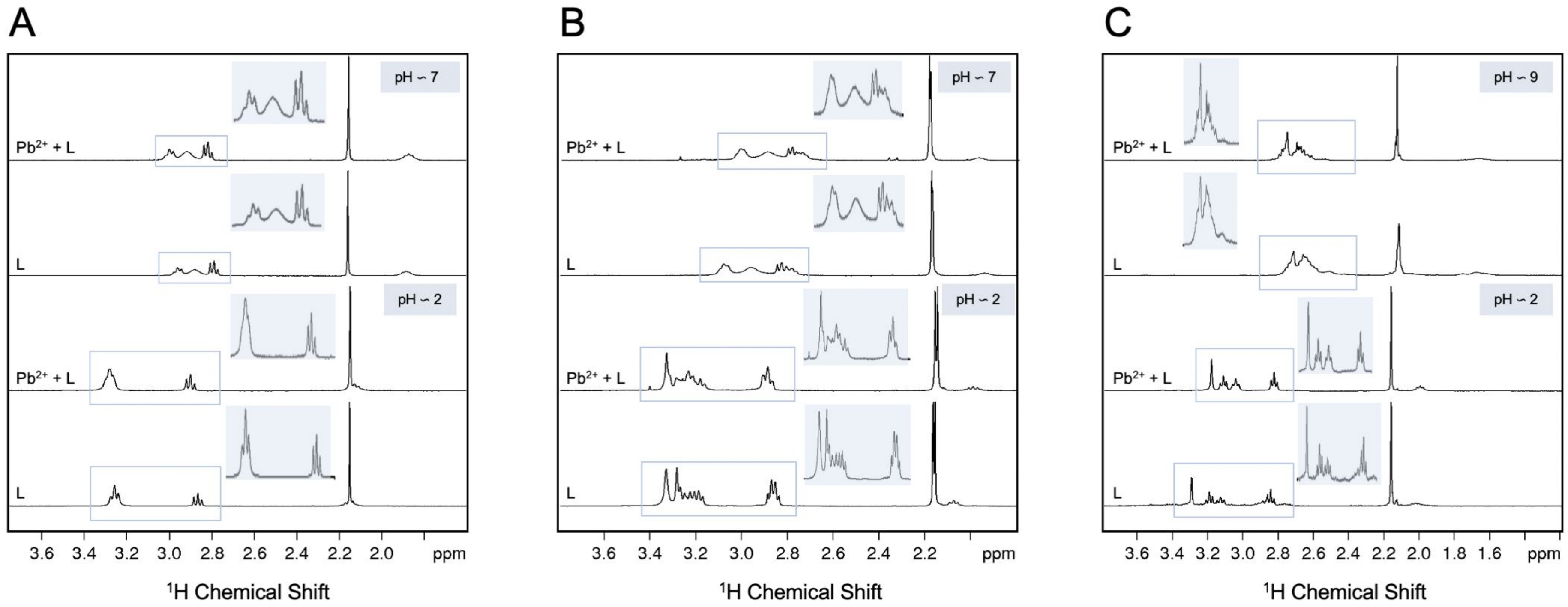

Figure S4. Representative ^{1}H-NMR spectra (400 MHz, T = 25 °C, 90 % H_2O + 10 % D_2O) of (A) Pb^{2+}-TACD3S, (B) Pb^{2+}-TRI4S and (C) Pb^{2+}-TE4S at different pH values ($C_{Pb^{2+}} = C_{ligand} = 1.0 \cdot 10^{-3}$ M). No complexation was observed as no spectral changes were detected over time respect to the free ligands (spectra of the free ligands were taken from ref.[1]).

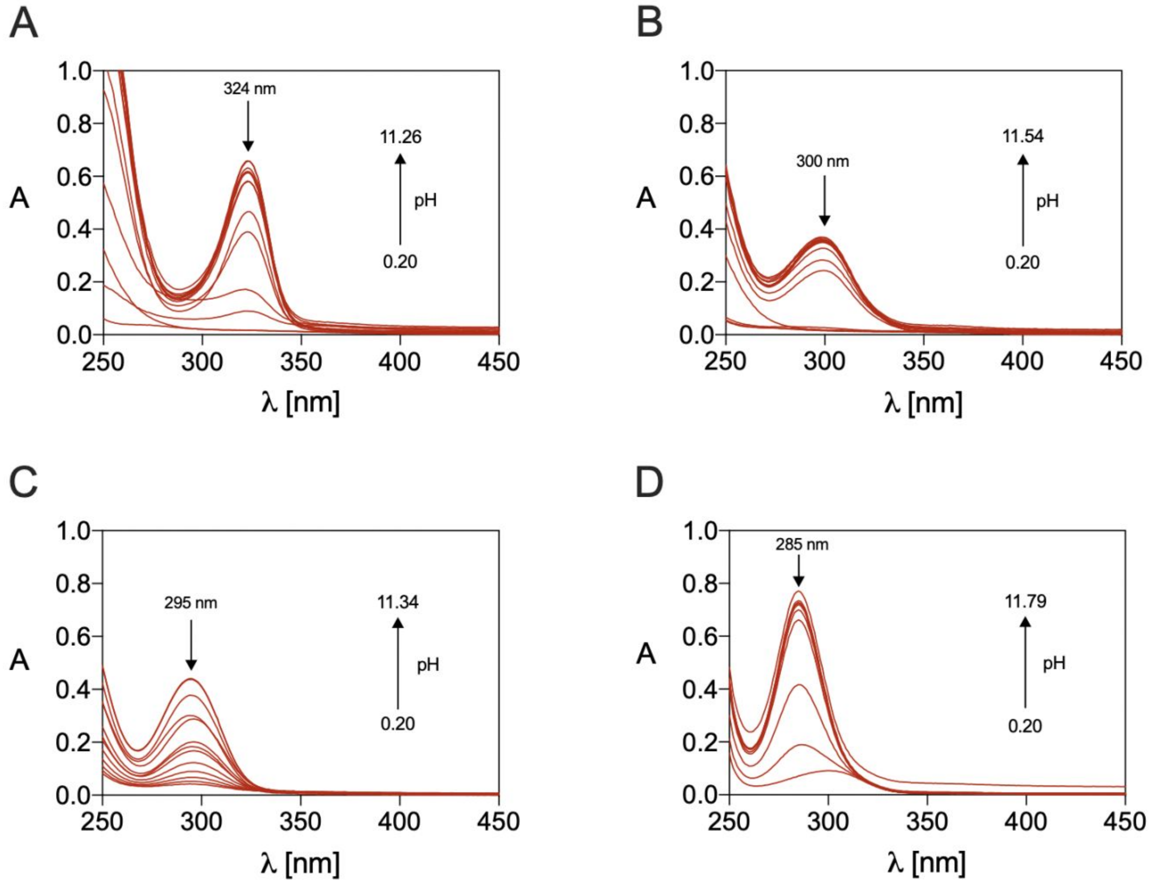

Figure S5. Representative electronic spectra of (A) Pb^{2+}-DO4S, (B) Pb^{2+}-DO3S, (C) Pb^{2+}-DO3SAm and (D) Pb^{2+}-DO2A2S at different pH ($C_{Pb^{2+}} = C_{ligand} = 1.0 \cdot 10^{-4}$ M, at T = 25 °C, I = 0.15 M $NaNO_3$).

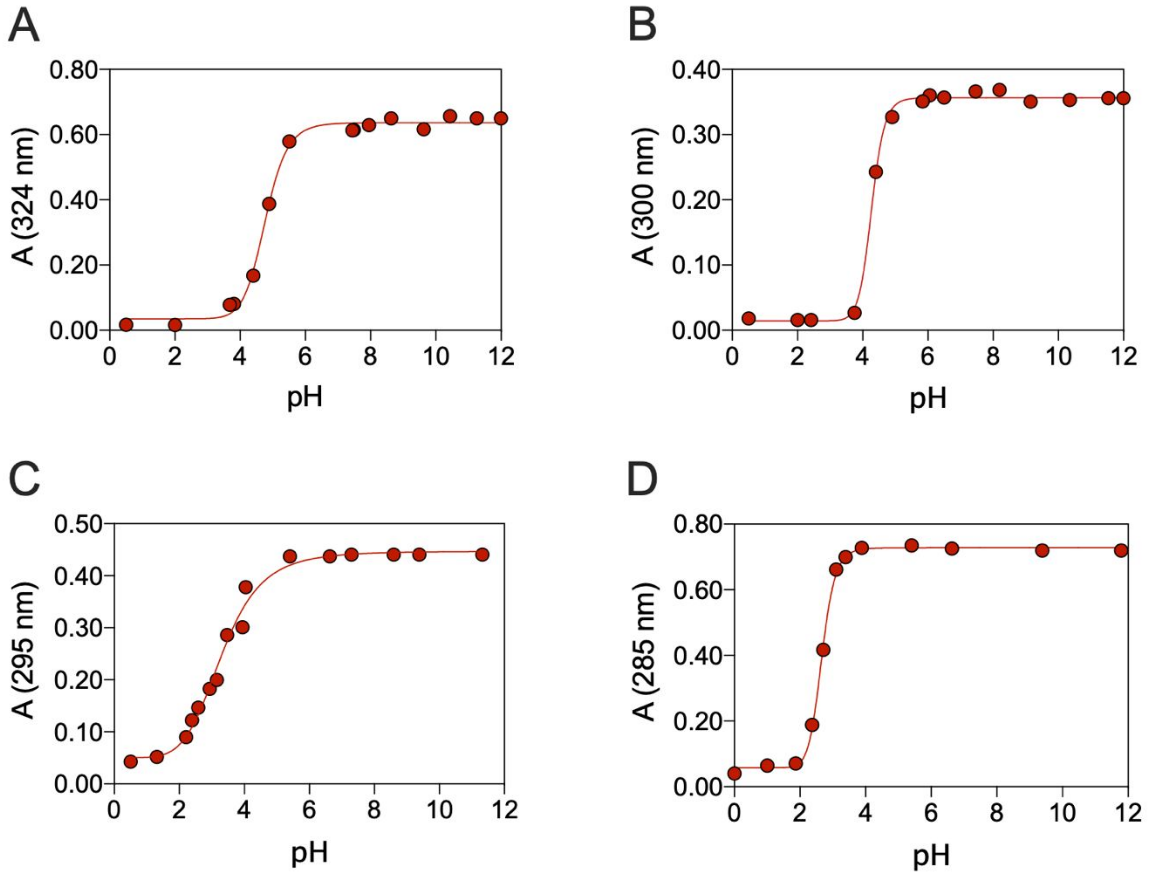

Figure S6. Representative experimental points and fitting line of $A(\lambda_{max})$ *vs.* pH for the UV-Vis spectrophotometric titration of the Pb^{2+} complexes with (A) DO4S, (B) DO3S (C) DO3SAm and (D) DO2A2S ($C_{Pb^{2+}}$ = C_{ligand} = $1.0 \cdot 10^{-4}$ M at T = 25 °C, I = 0.15 M $NaNO_3$). Data points were taken from **Figure S5**.

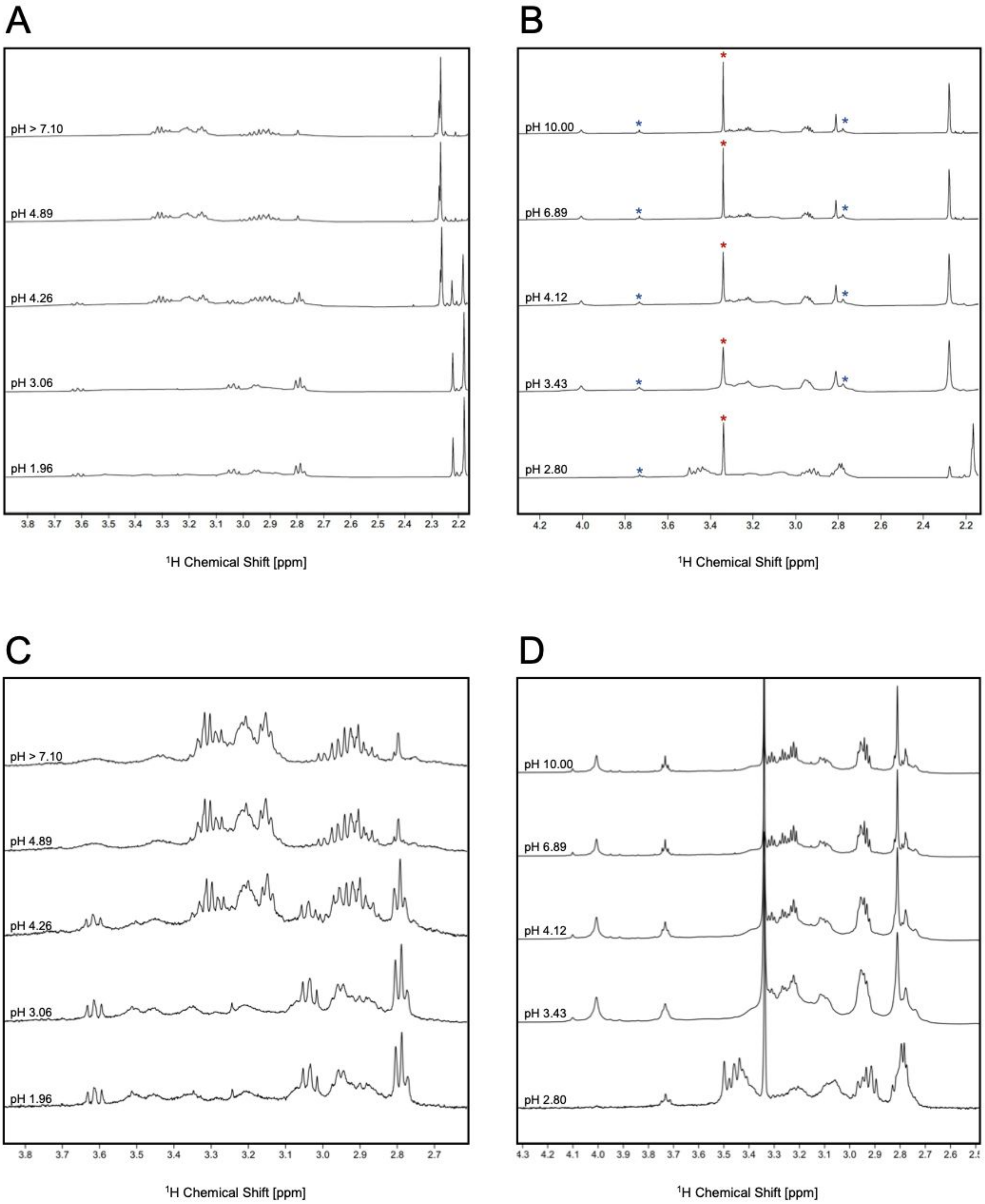

Figure S7. ^{1}H NMR spectra of (A) Pb^{2+}-DO3S (400 MHz, T = 25 °C, 90 % H_2O + 10 % D_2O, $C_{Pb^{2+}}$ = C_{DO3S} = $1.0 \cdot 10^{-3}$ M) and (B) Pb^{2+}-DO3SAm (600 MHz, T = 25 °C, 90 % H_2O + 10 % D_2O, $C_{Pb^{2+}}$ = C_{DO3SAm} = $8.0 \cdot 10^{-4}$ M) at different pH and (C, D) corresponding signals' enlargement in selected regions. The signals marked with blue asterisks have been tentatively attributed to impurities. The signal marked with red asterisks is related to residual methanol.

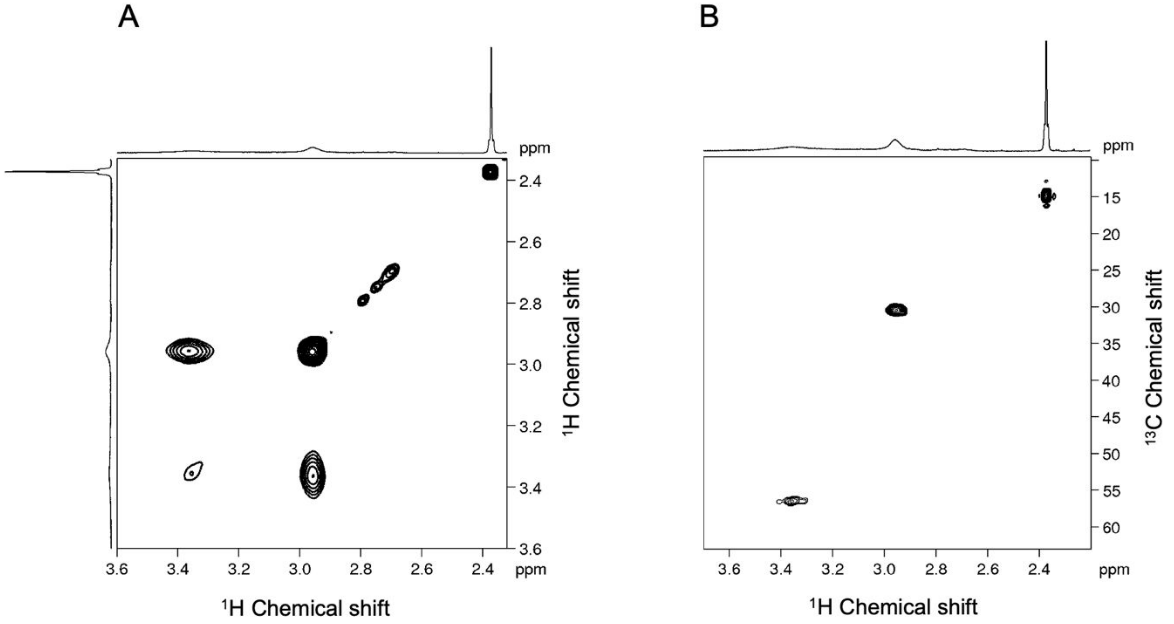

Figure S8. (A) ^{1}H-^{1}H TOCSY and (B) ^{1}H-^{13}C HSQC spectra of $[Pb(DO4S)]^{2+}$ (400 MHz, T = 25 °C, 90 % H_2O + 10 % D_2O, pH 8.56).

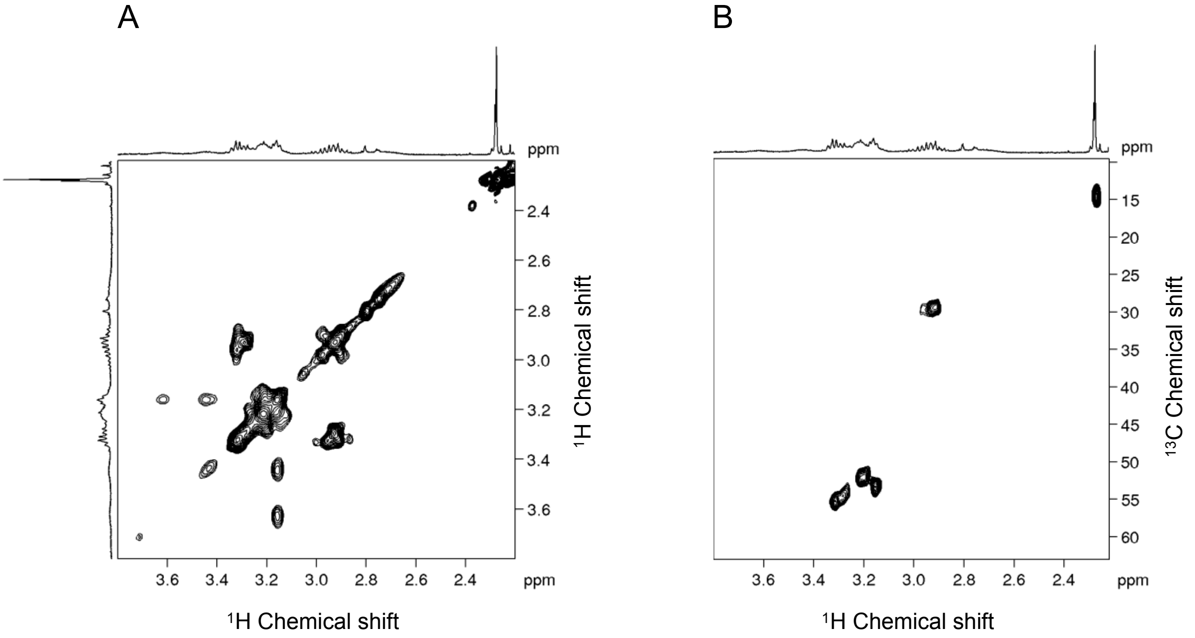

Figure S9. (A) ^{1}H-^{1}H TOCSY and (B) ^{1}H-^{13}C HSQC spectra of $[Pb(DO3S)]^{2+}$ (400 MHz, T = 25 °C, 90 % H_2O + 10 % D_2O, pH 9.57).

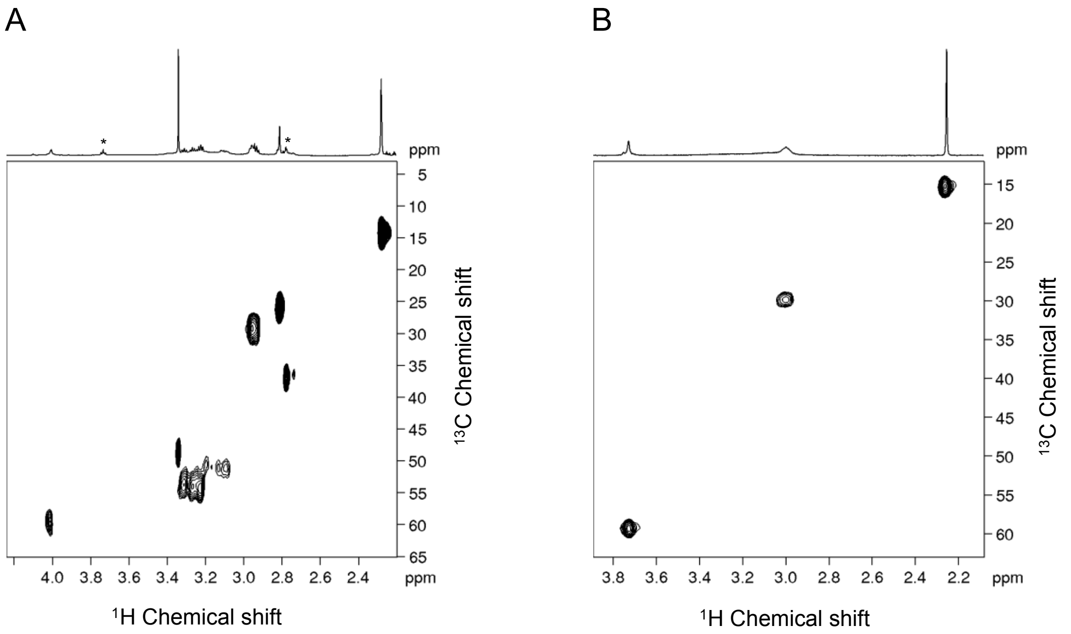

Figure S10. ^1H-^{13}C HSQC spectra of (A) $[Pb(DO3SAm)]^{2+}$ (600 MHz, T = 25 °C, 90 % H_2O + 10 % D_2O, pH 5.42) and (B) [Pb(DO2A2S)] (400 MHz, T = 25 °C, 90 % H_2O + 10 % D_2O, pH 8.09). The signals marked with asterisks have been tentatively attributed to impurities.

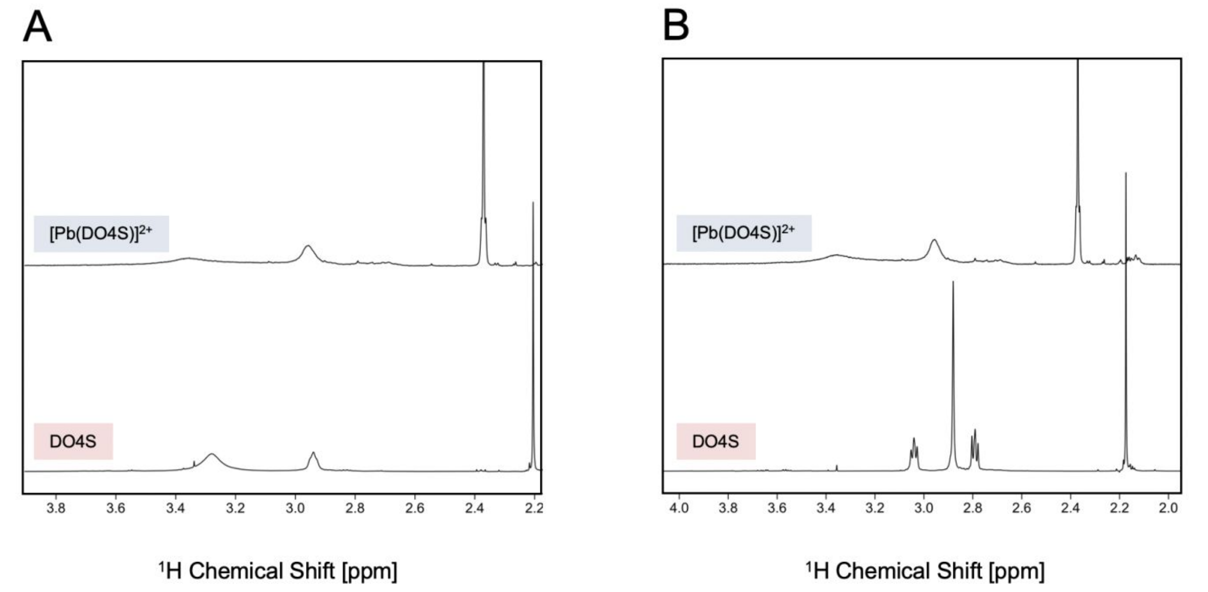

Figure S11. Comparison of the ^{1}H NMR spectra of free DO4S and $[Pb(DO4S)]^{2+}$ (400 MHz, T = 25 °C, 90 % H_2O + 10 % D_2O) at (A) pH 5 and (B) pH 9. Data for the free ligand were taken from ref.[2].

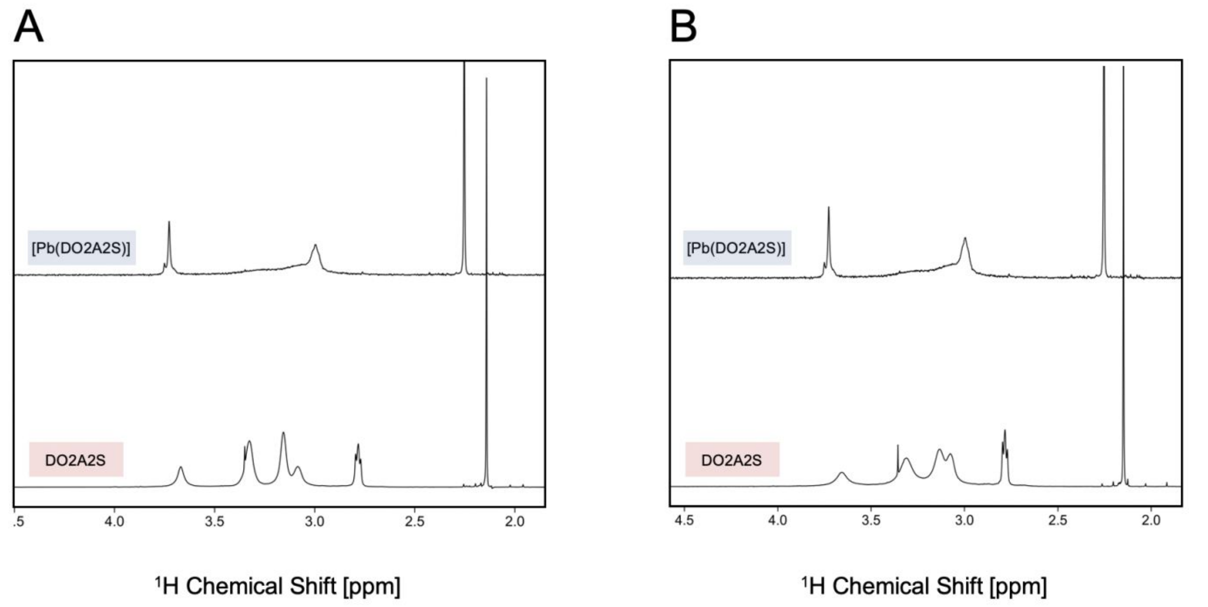

Figure S12. Comparison of the ^{1}H NMR spectra of free DO2A2S and [Pb(DO2A2S)] (400 MHz, T = 25 °C, 90 % H_2O + 10 % D_2O) at (A) pH 5 and (B) pH 9. Data for the free ligand were taken from ref.[2].

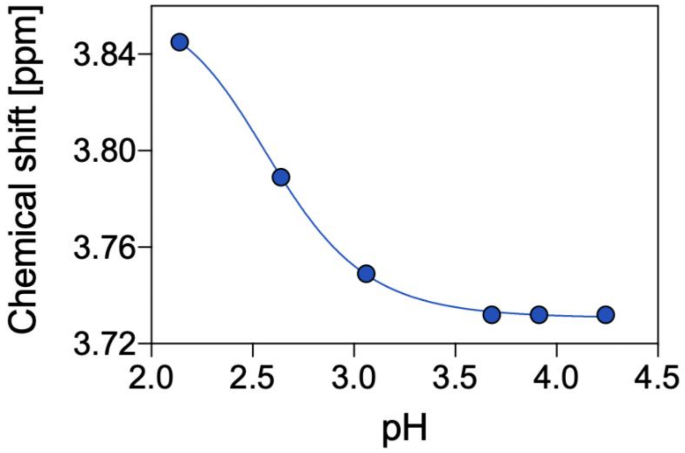

Figure S13. Variation of the chemical shift of the acetate protons of [Pb(DO2A2S)] as a function of pH (data were taken from **Figure 4** - **B**), and corresponding fitting line giving a pK_a value of 2.6 ± 0.1.

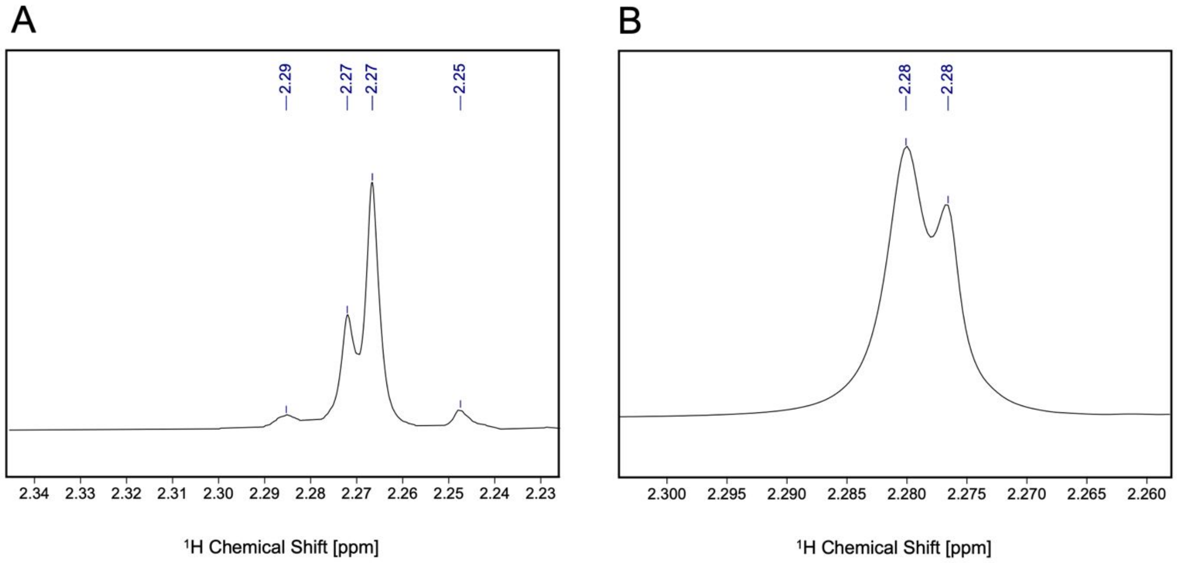

Figure S14. Enlargement of the SCH_3 region on the ^{1}H NMR spectra of (A) $[Pb(DO3S)]^{2+}$ and (B) $[Pb(DO3SAm)]^{2+}$ (data were taken from **Figure S7**).

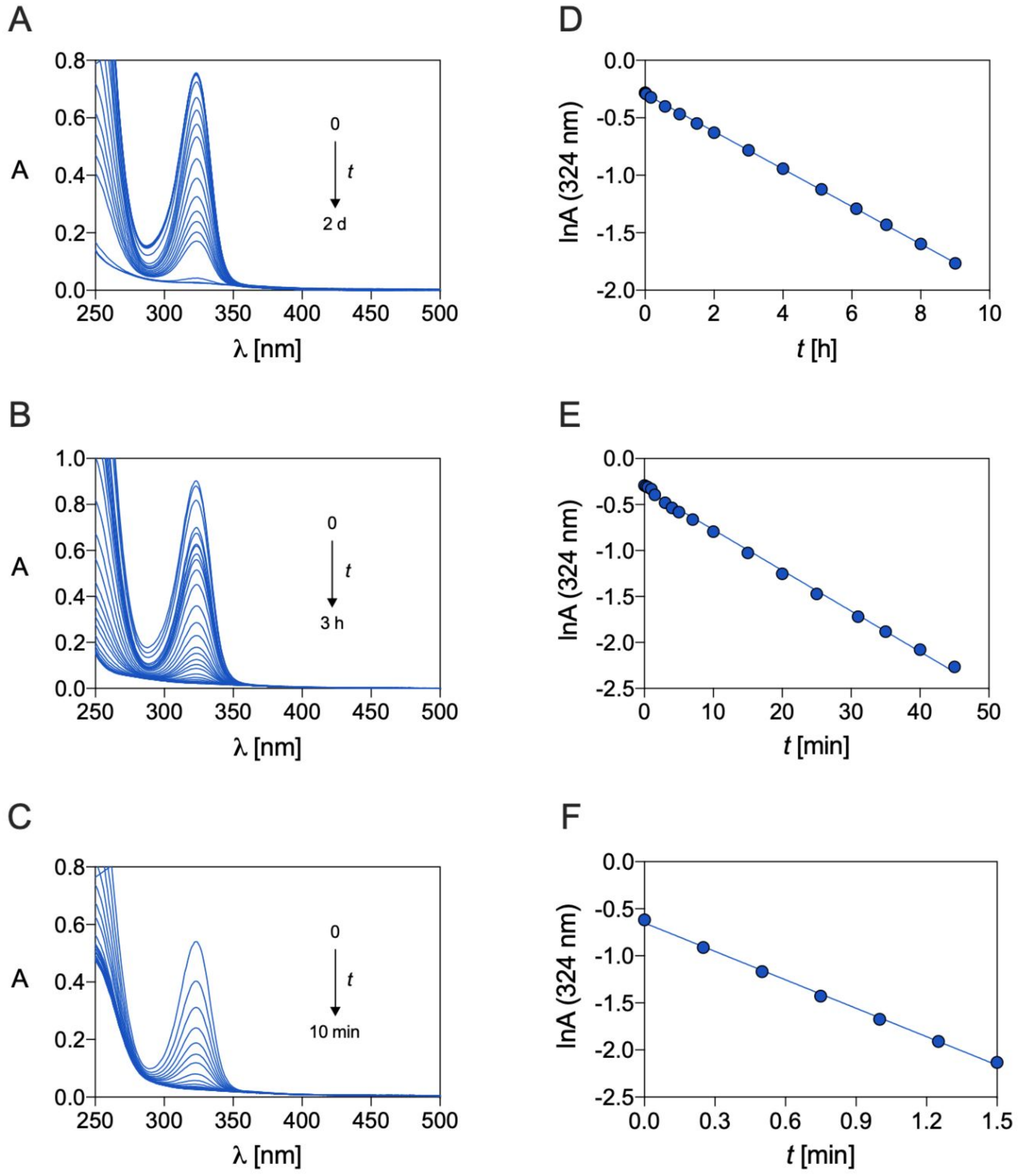

Figure S15. Representative variations of the UV-Vis spectra obtained during the acid decomplexation assays of $[Pb(DO4S)]^{2+}$ at (A) pH 2, (B) pH 1 and (C) pH 0; (D, E, F) ln*A*(324 nm) *vs*. *t*.

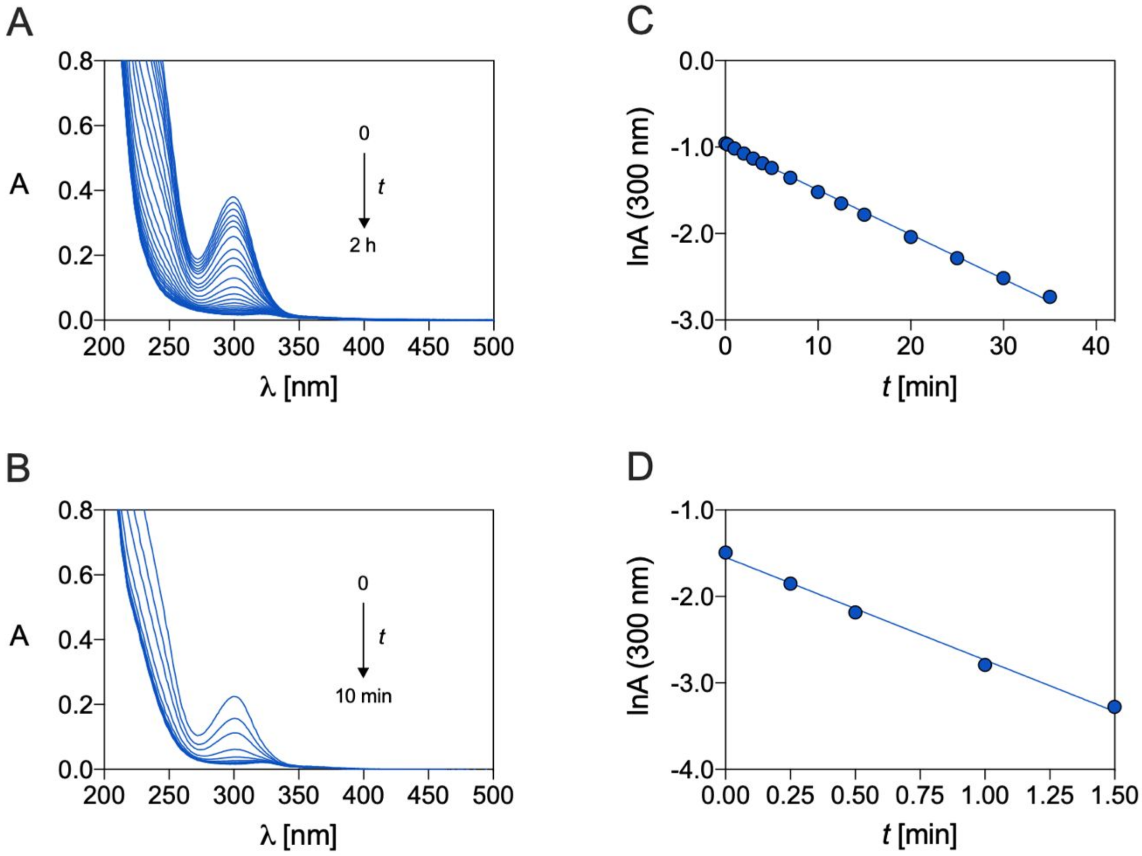

Figure S16. Representative variations of the UV-Vis spectra obtained during the acid decomplexation assays of $[Pb(DO3S)]^{2+}$ at (A) pH 2 and (B) pH 1; (D, E, F) ln*A*(300 nm) *vs*. *t*.

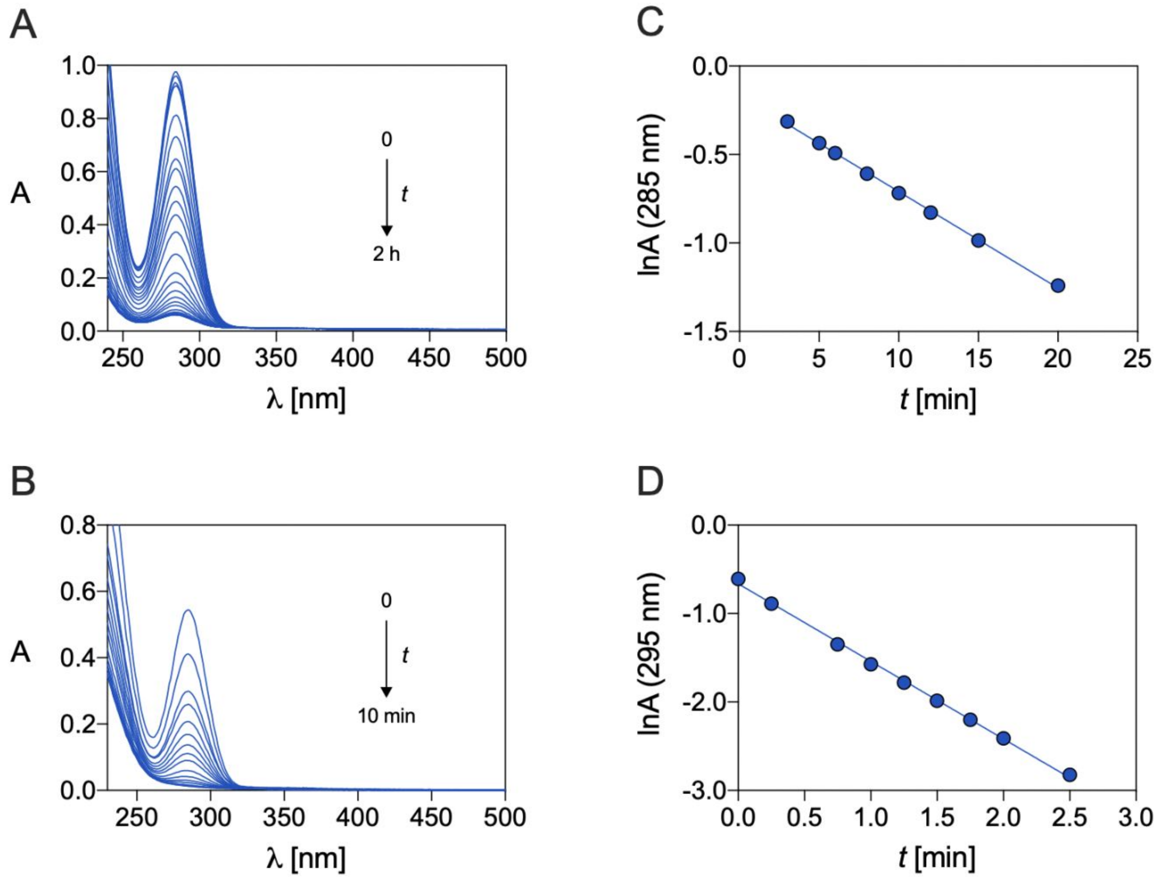

Figure S17. Representative variations of the UV-Vis spectra obtained during the acid decomplexation assays of [Pb(DO2A2S)] at (A) pH 2 and (B) pH 1; (D, E, F) ln*A*(295 nm) *vs*. *t*.

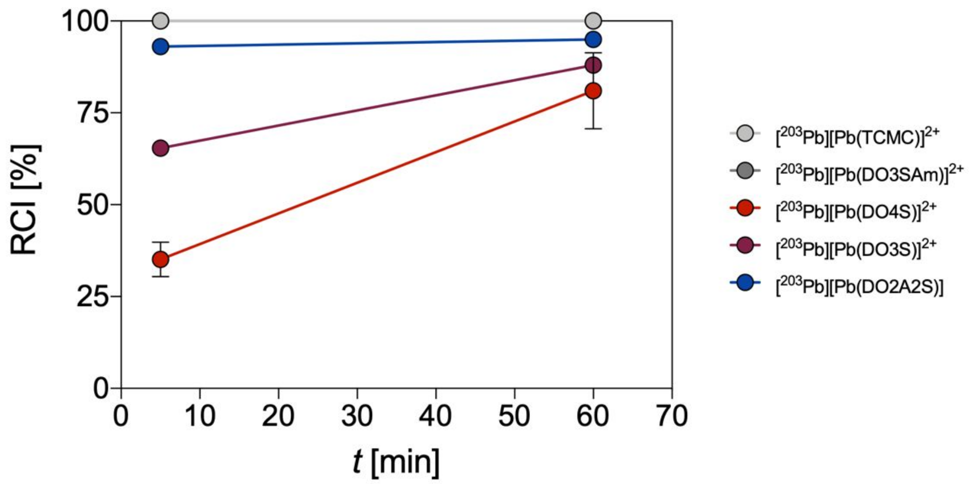

Figure S18. Time-dependent radiochemical incorporation of $[^{203}Pb]Pb^{2+}$ at room temperature for the investigated ligands (pH 7, $C_{ligand} = 10^{-4}$ M). Data for $[^{203}Pb][Pb(TCMC)]^{2+}$ and $[^{203}Pb][Pb(DO3SAm)]^{2+}$ are overlapped. For most of the points, the standard deviation bars were shorter than the height of the symbols, making them impossible to visualize (errors < 2%).

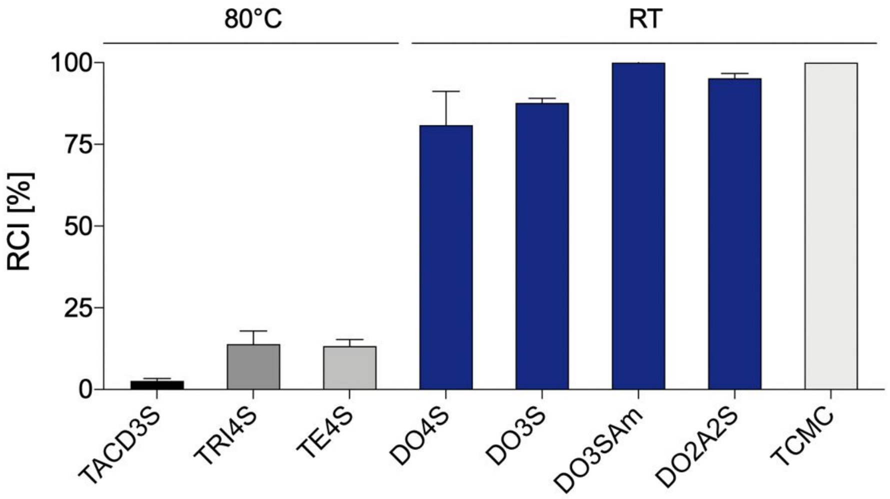

Figure S19. Comparison of the radiochemical incorporation of $[^{203}Pb][Pb(TACD3S)]^{2+}$, $[^{203}Pb][Pb(TRI4S)]^{2+}$, $[^{203}Pb][Pb(TE4S)]^{2+}$ and the cyclen-based chelators ($C_{ligand} = 10^{-4}$ M) at pH 7 after 1 h.

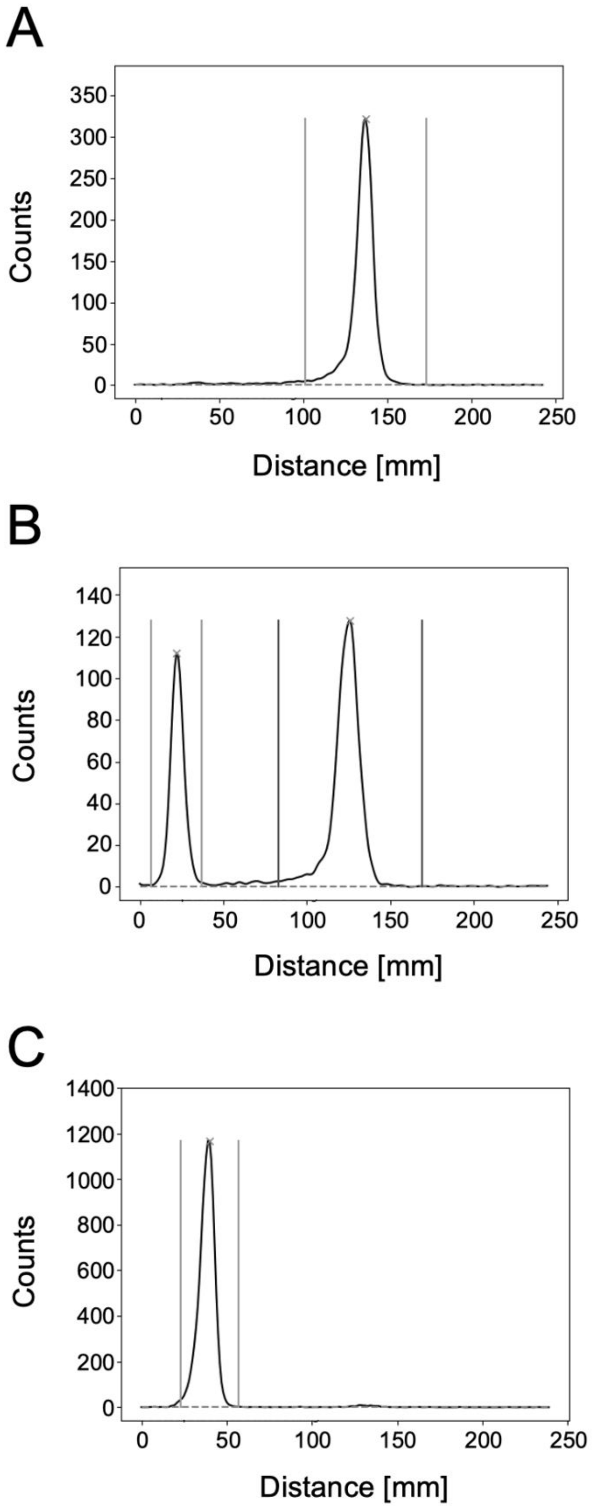

Figure S20. Representative iTLC radio-chromatograms of (A) unlabeled [^{203}Pb]Pb^{2+}, (B) non-quantitative labelling reaction and (C) quantitative labelling reaction.

Supplementary Tables

Table S1. Time required to reach the equilibrium during the Pb^{2+} complexes formation reactions at various pH with DO4S, DO3S, DO3SAm and DO2A2S.

pH	$t_{equilibrium}$			
	**DO4S**	**DO3S**	**DO3SAm**	**DO2A2S**
2.0	(a)	(a)	(a)	~ 3 h (b)
3.7	(a)	(a)	–	~ 20 min
5.0	~ 24 h (b)	~ 6 h (b)	~ 2 h	< 10 s (c)
7.4	~ 1 h	~ 3 min	< 10 s (c)	< 10 s (c)

(a) No complex formation
(b) Complexation not quantitative
(c) Complex formation during the mixing time of the two reagents

Table S2. Electronic properties (λ_{max} and ε) of the Pb^{2+} complexes with DO4S, DO3S, DO3SAm and DO2A2S at T = 25 °C.

**Complex**	**Pb^{2+}-DO4S**	**Pb^{2+}-DO3S**	**Pb^{2+}-DO3SAm**	**Pb^{2+}-DO2A2S**
**λ_{max} [nm]**	324	300	295	285
**ε [L/cm·mol]**	$6.5 \cdot 10^3$	$4.5 \cdot 10^3$	$4.0 \cdot 10^3$	$7.0 \cdot 10^3$

Table S3. Chemical shift, multiplicity, area, and ^1H/^{13}C-NMR resonance assignments for the Pb^{2+}-complexes with DO4S, DO3S, DO3SAm and DO2A2S at T = 25 °C.

**Complex**	**^1H**			**^{13}C**	**Assignation**
	**δ [ppm]**	**Multiplicity**	**Area**	**δ [ppm]**	
**$[Pb(DO4S)]^{2+}$**	2.37	s	12	14.94	SCH_3
	2.96	m br	8	30.49	SCH_2
	3.04 - 3.70	m br	24	56.72	NCH_2 ring + arms
**$[Pb(DO3S)]^{2+}$**	2.27	s	6	14.61	SCH_3
	2.28	s	3		SCH_3
	2.77 - 3.02	m	6	29.61	SCH_2
	3.07-3.26 + 3.39-3.70	m	16	52.3 - 55.15	NH_2 ring
	3.27 - 3.37	m	6		NCH_2 arms
**$[Pb(DO3SAm)]^{2+}$**	2.28	s	9	14.5	SCH_3
	2.81	s	3	2.81	CONH<u>CH_3</u>
	2.95	m	6	2.95	SCH_2
	3.06 - 3.38	m	22	51.1 - 53.6	NCH_2
	4.00	s	2	59.8	-<u>CH_2</u>CO
**[Pb(DO2A2S)]**	2.25	s	6	15.4	SCH_3
	3.00	m	4	30.1	SCH_2
	2.65 - 3.40	m br	20	(*)	NCH_2 ring + arms
	3.72	s	4	59.5	CH_2COOH

s = singlet; m = multiplet; br = broad

(*) The extremely broadness of the signal prevented its detection

Table S4. Chemical shift, multiplicity, area, and ^{1}H resonance assignments for $[Pb(DO4S)]^{2+}$ and [Pb(DO2A2S)] at T = 65 °C.

Complex	δ [ppm]	Multiplicity	Area	Assignation
**$[Pb(DO4S)]^{2+}$**	2.37	s	12	SCH_3
	2.97	t	8	SCH_2
	3.11 - 3.23	m br	8	NCH_2 ring
	3.3 - 3.34	m br	8	NCH_2 ring
	3.34 - 3.43	m br	8	NCH_2 arms
**[Pb(DO2A2S)]**	2.26	s	6	SCH_3
	2.98	t	4	SCH_2
	3.02 - 3.23	m br	16	NCH_2 ring
	3.29	t	4	NCH_2 arms
	3.70	s	4	CH_2COOH

s = singlet; m = multiplet; t = triplet; br = broad

Supplementary References

(1) Tosato, M.; Pelosato, M.; Franchi, S.; Isse, A. A.; May, N. V.; Zanoni, G.; Mancin, F.; Pastore, P.; Badocco, D.; Asti, M.; Di Marco, V. When Ring Makes the Difference: Coordination Properties of Cu^{2+}/Cu^{+} Complexes with Sulfur-Pendant Polyazamacrocycles for Radiopharmaceutical Applications. *New J. Chem.* **2022**, *46*, 10012-10025.

(2) Tosato, M.; Verona, M.; Doro, R.; Dalla Tiezza, M.; Orian, L.; Andrighetto, A.; Pastore, P.; Marzaro, G.; Di Marco, V. Toward Novel Sulphur-Containing Derivatives of Tetraazacyclododecane: Synthesis, Acid-Base Properties, Spectroscopic Characterization, DFT Calculations, and Cadmium(II) Complex Formation in Aqueous Solution. *New J. Chem.* **2020**, *44*, 8337-8350.
